# Avian Hepatitis E Virus ORF2 Protein Interacts with Rap1b to Induce Cytoskeleton Rearrangement That Facilitates Virus Internalization

**DOI:** 10.1128/spectrum.02265-21

**Published:** 2022-02-09

**Authors:** Beibei Zhang, Mengnan Fan, Jie Fan, Yuhang Luo, Jie Wang, Yajing Wang, Baoyuan Liu, Yani Sun, Qin Zhao, Julian A. Hiscox, Yuchen Nan, En-Min Zhou

**Affiliations:** a Department of Preventive Veterinary Medicine, College of Veterinary Medicine, Northwest A&F University, Yangling, Shaanxi, China; b Institute of Infection, Veterinary and Ecological Sciences, University of Liverpool, Liverpool, United Kingdom; Wuhan Institute of Virology

**Keywords:** hepatitis E virus, Rap1b, virion internalization, cytoskeleton rearrangement, viral capsids

## Abstract

Avian hepatitis E virus (HEV) causes liver diseases and multiple extrahepatic disorders in chickens. However, the mechanisms involved in avian HEV entry remain elusive. Herein, we identified the RAS-related protein 1b (Rap1b) as a potential HEV-ORF2 protein interacting candidate. Experimental infection of chickens and cells with an avian HEV isolate from China (CaHEV) led to upregulated expression and activation of Rap1b both *in vivo* and *in vitro*. By using CaHEV capsid as mimic of virion to treat cell *in vitro*, it appears that the interaction between the viral capsid and Rap1b promoted cell membrane recruitment of the downstream effector Rap1-interacting molecule (RIAM). In turn, RIAM further enhanced Talin-1 membrane recruitment and retention, which led to the activation of integrin α5/β1, as well as integrin-associated membrane protein kinases, including focal adhesion kinase (FAK). Meanwhile, FAK activation triggered activation of downstream signaling molecules, such as Ras-related C3 botulinum toxin substrate 1 RAC1 cell division cycle 42 (CDC42), p21-activated kinase 1 (PAK1), and LIM domain kinase 1 (LIMK1). Finally, F-actin rearrangement induced by Cofilin led to the formation of lamellipodia, filopodia, and stress fibers, contributes to plasma membrane remodeling, and might enhance CaHEV virion internalization. In conclusion, our data suggested that Rap1b activation was triggered during CaHEV infection and appeared to require interaction between CaHEV-ORF2 and Rap1b, thereby further inducing membrane recruitment of Talin-1. Membrane-bound Talin-1 then activates key Integrin-FAK-Cofilin cascades involved in modulation of actin kinetics, and finally leads to F-actin rearrangement and membrane remodeling to potentially facilitate internalization of CaHEV virions into permissive cells.

**IMPORTANCE** Rap1b is a multifunctional protein that is responsible for cell adhesion, growth, and differentiation. The inactive form of Rap1b is phosphorylated and distributed in the cytoplasm, while active Rap1b is prenylated and loaded with GTP to the cell membrane. In this study, the activation of Rap1b was induced during the early stage of avian HEV infection under the regulation of PKA and SmgGDS. Continuously activated Rap1b recruited its effector RIAM to the membrane, thereby inducing the membrane recruitment of Talin-1 that led to the activation of membrane α5/β1 integrins. The triggering of the signaling pathway-associated Integrin α5/β1-FAK-CDC42&RAC1-PAK1-LIMK1-Cofilin culminated in F-actin polymerization and membrane remodeling that might promote avian HEV virion internalization. These findings suggested a novel mechanism that is potentially utilized by avian HEV to invade susceptible cells.

## INTRODUCTION

Hepatitis E virus (HEV), which is a positive-sense, single-stranded RNA virus that causes both acute and chronic hepatitis in humans, belongs to the genus *Orthohepevirus* within the family *Hepeviridae* (1). Currently, the range of hosts susceptible to HEV and newly identified HEV isolates is expanding. In the latest HEV classification scheme, the family *Hepeviridae* comprises two genera, namely, *Orthohepevirus* (including all mammalian and avian HEV isolates) and *Piscihepevirus* (including only trout HEV isolates). Within the genus *Orthohepevirus*, different virus isolates are further categorized into species based on host tropisms and genome sequences (2). HEV genotypes infecting humans include two anthropotropic genotypes (HEV-1 and HEV-2) and two zoonotic genotypes (HEV-3 and HEV-4), all of which belong to the species *Orthohepevirus A* (3). Moreover, species B, C, and D of *Orthohepevirus* encompass isolates that were originally believed to infect only nonhuman mammalian and avian species (2, 3), whereas recent findings suggest that *Orthohepevirus* C may be zoonotic (4). Avian HEV, which is a member of the species *Orthohepevirus* B, causes large liver and spleen (BLS) disease in chickens, which has adversely impacted the poultry industry (1). The genome of avian HEV is approximately 6.6 kb in length and relatively shorter in length than mammalian HEV (5), although phylogenetic analysis indicates that avian HEV is genetically less related to other known HEV strains (6). Notably, experimental infections of avian HEV in mammalian hosts, such as rabbits, have confirmed that HEVs can jump host species (7). Although no zoonotic avian HEV capable of infecting humans has yet been detected, experimental evidence suggests a cross-species transmission of avian HEV threat to mammalian hosts, as our previous report demonstrated that avian HEV could infect SPF rabbits experimentally (7).

Currently, three well-characterized open reading frames (ORFs) have been identified in all HEV genomes regardless of species tropism or genotype. The HEV-ORF1 protein, which is translated from the mRNA-like genome of HEV, encodes all viral replicases required for viral RNA replication (8, 9). HEV-ORF2 and ORF3 overlap with each other and are translated from viral subgenomic mRNAs generated during viral RNA replication (10). A protease-cleaved shorter form of full-length HEV-ORF2 protein forms the viral capsid protein, the major constituent of the HEV virion. Moreover, the HEV-ORF3 protein appears to be a class I viroporin that is essential for HEV virion release and biogenesis of quasi-enveloped virions (11).

In mammalian HEVs, the full-length ORF2 protein contains 660 amino acids (aa) and is responsible for viral infectivity and the induction of host protective immune responses (12). Although the identities of cellular receptors involved in HEV infection remain elusive, HEV capsids consisting of HEV-ORF2 contain β-barrel fold structures and are predicted to have polysaccharide-binding sites that appear to be involved in cell-receptor binding and capsid disassembly (13, 14). Moreover, avian HEV-ORF2 encodes a protein with a mass of ∼67 kDa that appears to possess the same function (5). The immunogenic cross-reactivity of ORF2s of avian, pig, and human HEVs has been reported to likely reflect multiple shared epitopes among various HEV genotypes (15). Therefore, truncated ORF2 proteins produced using various recombinant expression systems could potentially serve as components of cross-species vaccines (16, 17). In addition to its potential use as a vaccine, recombinant HEV-ORF2 protein has served as a probe for use in identifying potential HEV cellular receptors. Several host proteins have been shown to interact with HEV-ORF2 within cells, including heat shock protein 90, GRP78 (18), asialo-glycoprotein receptor (ASGPR), organic anion-transporting polypeptide 1A2 (OATP1A2), retinol-binding protein 4 (RBP4), and heparan sulfate proteoglycans (HSPGs) (17, 19–21), which play important roles during HEV attachment and internalization.

In our previous studies, a truncated avian HEV-ORF2 protein (ap237), which is homologous to truncated human HEV-p239 and capable of forming virus-like particles (VLPs) (19), was employed as a bait to probe for potential binding proteins from avian liver tissue (19), whereas OATP1A2 was identified as a CaHEV capsid-interacting protein that was shown to promote HEV replication in the avian hepatocarcinoma cell line LMH (19). In the present study, RAS-related protein 1b (Rap1b), another potential ap237-interacting protein, was identifed and investigated for its role in avian HEV infection. Our data suggested that Rap1b was upregulated and activated during CaHEV infection both *in vivo* and *in vitro*. By employing the ap237 protein to mimic the CaHEV capsid protein, we obtained data indicating that the interaction between Rap1b and ap237 protein enhanced membrane recruitment of Rap1-interacting molecule (RIAM), a downstream effector of Rap1b (22). In turn, RIAM membrane recruitment led to the activation of a signaling cascade downstream of Rap1b/RIAM, which resulted in membrane retention of Talin-1 (23). Consequently, membrane-bound Talin-1 acted as an integrin activation protein to activate membrane α5/β1 integrins, which resulted in cytoskeletal rearrangement that might promote CaHEV internalization by permissive cells.

## RESULTS

### Rap1b, a CaHEV-ORF2-interacting protein, is upregulated and activated during CaHEV infection *in vivo*.

**FIG 1:**
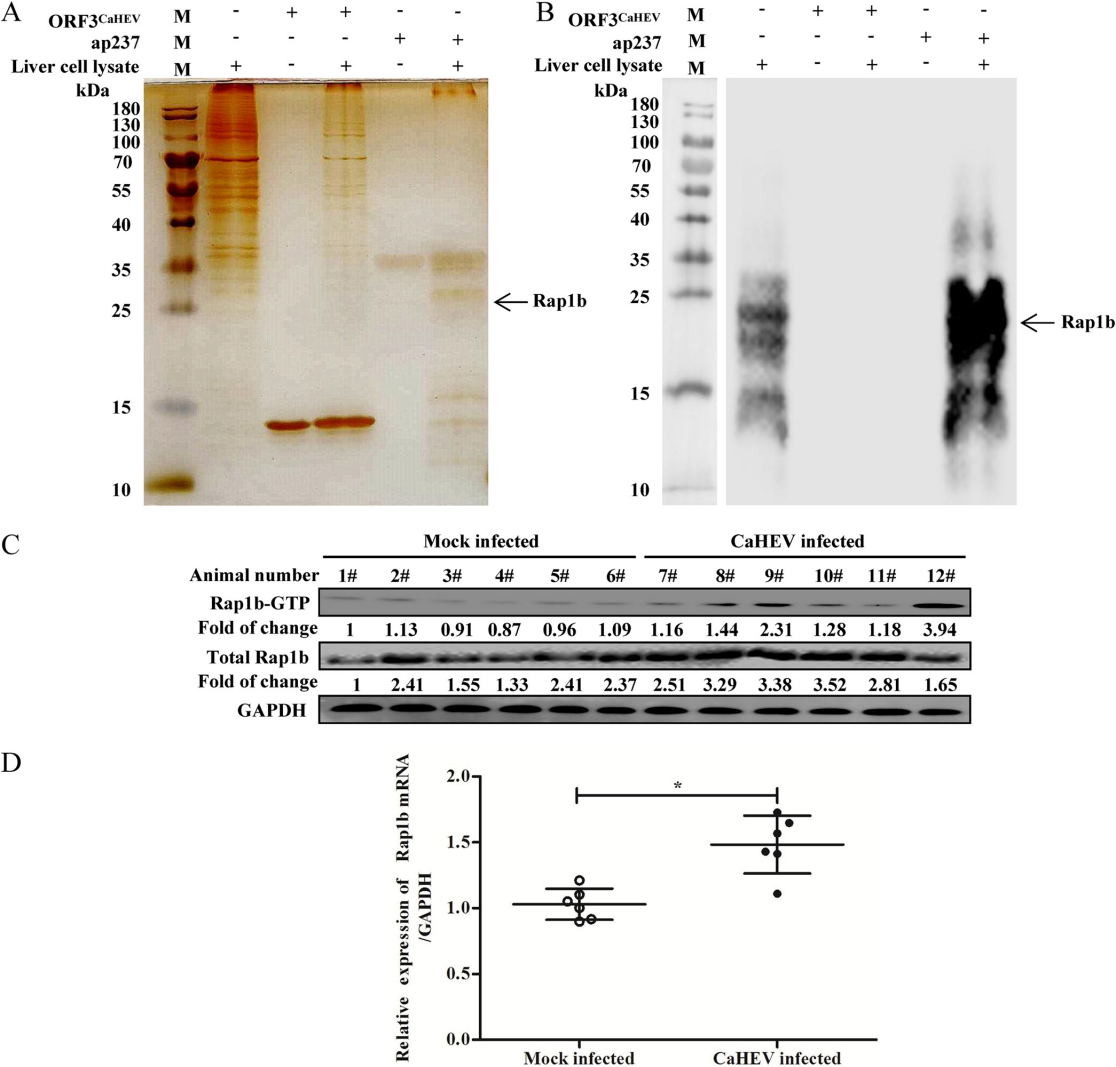
Rap1b is involved in avian HEV infection. (A) Sepharose 4B resin conjugated with ap237 protein was used to pull down the potential interacting candidates from chicken liver cell lysates. After SDS–PAGE, ap237 interacting proteins were visualized by silver staining. Sepharose 4B resin conjugated with recombinant ORF3^CaHEV^ protein was included as a control. (B) Samples harvested from the pulldown assay were subjected to Western blotting using an anti-Rap1b antibody. (C) In total, 12 SPF chickens were divided into 2 groups (*n* = 6): one group was inoculated with CaHEV, and the other group was inoculated with PBS. Liver samples harvested at 42 days postinoculation (dpi) were homogenized for analysis of Rap1b and Rap1b-GTP levels using Western blotting. GAPDH was probed from the same sample as a control to normalize the total protein load. (D) Homogenized chicken liver samples were harvested for reverse transcription and qPCR to evaluate Rap1b mRNA levels. The housekeeping gene GAPDH was used as a control to normalize the total RNA input. Error bars represent the variation of data obtained from 6 chicken liver samples. Statistical significance between the indicated groups is marked as *, *P < *0.05.

In our investigation for reanalysis of mass spectrometry (MS) data originally detecting the interaction between CaHEV capsid protein (ap237) and OATP1A2 (19), Rap1b was identified as another interacting partner of ap237. To confirm this observation, a pulldown assay was carried out again to probe chicken liver tissue samples with ap237 protein as bait. SDS–PAGE and silver staining revealed Rap1b within liver cell lysates with a predicted molecular weight consistent with MS (Fig. 1A). Next, a Rap1b-specific antibody was employed to probe the pull-down sample, and Rap1b was detected as an ap237-interacting host protein (Fig. 1B), which suggests that Rap1b interacted with the CaHEV capsid during the pulldown assay. Moreover, to further examine the potential connection between Rap1b and avian HEV infection, liver tissue samples from Mock-infected (*n* = 6) and CaHEV-infected (*n* = 6) chickens were collected for transcription- and translation-level assessments of Rap1b expression. Our data demonstrated that CaHEV infection significantly promoted the expression of Rap1b protein in chicken liver samples (Fig. 1C), with mRNA expression results aligning with Rap1b protein expression results obtained from CaHEV-infected chickens (Fig. 1D). As a GTPase, Rap1b activation depends on guanine nucleotide exchange factors (GEFs) that promote the binding of Rap1b with GTP to form Rap1b-GTP (24). Our data demonstrated that Rap1b activation (based on Rap1b-GTP level) was significantly upregulated in CaHEV-infected chickens (Fig. 1C), thereby indicating that enhanced activation of Rap-1b occurred during CaHEV infection *in vivo*. Therefore, these data suggested that Rap1b, a potential CaHEV capsid-interacting host protein, may involve in CaHEV infection *in vivo*.

### CaHEV infection-induced Rap1b activation and upregulation require a direct interaction between Rap1b and ORF2 protein.

**FIG 2:**
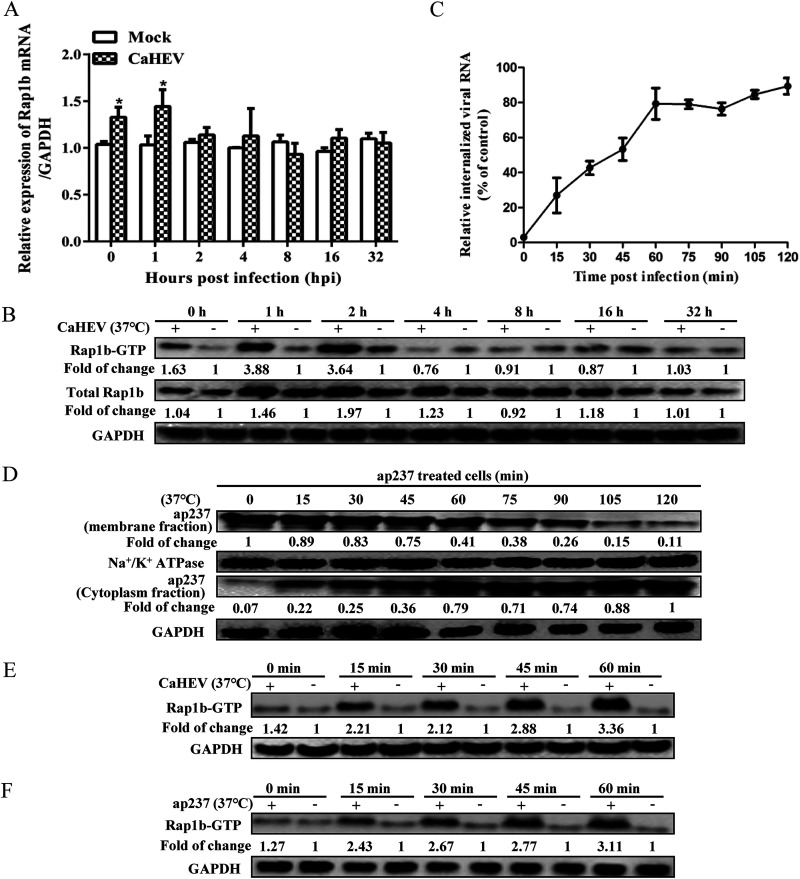
CaHEV promotes upregulation and activation of Rap1b at the early stage of infection. (A) LMH-1A2 cells were incubated with CaHEV at 4°C for 2 h to allow sufficient virion attachment and were then transferred to 37°C for 1 h to trigger virion internalization, followed by harvesting at the indicated time points to evaluate Rap1b mRNA by qPCR. Error bars represent the variation from at least three independent experiments. Statistical significance between the Mock and infected group is marked as *, *P* <0.05. (B) LMH-1A2 cells were infected with CaHEV using the same procedure as in Panel A, and then cells from different groups were harvested at the indicated time points to evaluate Rap1b and Rap1b-GTP levels using Western blotting. (C) LMH-1A2 cells were incubated with CaHEV at 4°C for 2 h to allow virion attachment and then transferred to 37°C for different times for virion internalization. Noninternalized virions were removed by treating the cells with citrate buffer for 30 s. Then, the cells were harvested for qPCR analysis of internalized viral RNA. The viral RNA level in LMH-1A2 cells incubated with CaHEV at 4°C for 2 h without further treatment was set as 100%. Error bars represent variation from at least three independent experiments. (D) LMH-1A2 cells were incubated with ap237 protein at 4°C for 1 h to allow sufficient attachment, and unbound protein was removed by washing the cells with precooled PBS. Then, the cells were transferred to 37°C for different times as indicated to trigger ap237 protein internalization. The membrane and cytoplasmic fractions from the same group of cells were separated and subjected to Western blotting to evaluate internalized ap237 protein. The Na^+^/K^+^ ATPase protein and GAPDH were used as controls to confirm the complete separation of membrane/cytoplasmic fractions from the same sample. (E) LMH-1A2 cells were incubated with CaHEV at 4°C for 2 h to allow sufficient virion attachment and then transferred to 37°C for different times (0, 15, 30, 45, and 60 min) to trigger virion internalization, followed by harvesting for evaluation of Rap1b-GTP levels using Western blotting. (F) LMH-1A2 cells were incubated with ap237 protein at 4°C for 1 h to allow protein attachment and then transferred to 37°C for different times (0, 15, 30, 45, and 60 min) to trigger protein internalization, followed by harvesting of the cells for evaluation of Rap1b-GTP levels by Western blotting.

Since the aforementioned *in vivo* data suggested that Rap1b could be upregulated and activated during CaHEV infection, *in vitro* studies using hepatocellular carcinoma LMH cells with stable expression of OATP1A2 (LMH-1A2) were conducted to comprehensively verify the *in vivo* results. LMH-1A2 cells were inoculated with CaHEV at 4°C to allow sufficient virion attachment, followed by transfer of the cells to 37°C to trigger endocytosis-mediated virion internalization and initiate CaHEV infection. Our data demonstrated that mRNA encoding Rap1b protein was upregulated after the virus-binding stage (0 min) and reached peak levels at 1 h after the induction of internalization by a temperature switch to 37°C, whereas similar levels of Rap1b mRNA between normal and CaHEV-infected cells persisted at later time points (Fig. 2A). Moreover, consistent with the *in vivo* results, significant elevation of both the total Rap1b protein level and activated Rap1b (Rap1b-GTP) was observed within 1 h of the temperature switch to 37°C (Fig. 2B), with Rap1b-GTP reaching peak levels within 1 h of the temperature switch that then decreased (Fig. 2B), whereas the total Rap1b level reached peak levels within 2 h of the temperature switch (Fig. 2B). Concurrently, qPCR-based viral RNA quantification was conducted to estimate internalized CaHEV viral particles. The results suggested that rapid internalization of virions occurred between 45 min and 60 min after the temperature switch and then plateaued after 60 min, with the internalization of the majority (over 80%) of attached virions occurring within 2 h of the temperature switch (Fig. 2C). These results suggested that virion attachment and internalization triggered upregulation and activation of Rap1b at early stages of CaHEV infection.

The above-mentioned results suggested that an interaction between the HEV capsid and Rap1b was required to induce subsequent Rap1b upregulation and activation. However, since CaHEV infection in LHM-1A2 cells was not sufficient for us to evaluate the internalization of viral capsids at the protein level (Western blotting), recombinant ap237 protein was employed as a mimic of CaHEV capsid for LMH-1A2 cells. Consistent with the internalized viral RNA levels, triggering endocytosis by a temperature switch to 37°C rapidly induced ap237 protein internalization, as indicated by decreasing ap237 protein levels in the membrane fraction in step with increasing ap237 protein levels in the cytoplasmic fraction (Fig. 2D). As Rap1b activation is the key trigger of downstream signaling, the activation kinetics of Rap1b were next examined after the addition of CaHEV or ap237 protein to LMH-1A2 cells. As demonstrated in Fig. 2E and F, Rap1b activation patterns were similar between CaHEV-infected and ap237-inoculated LMH-1A2 cells, whereby the moment of Rap1b activation in both systems basically coincided with the moment of virus entry.

**FIG 3:**
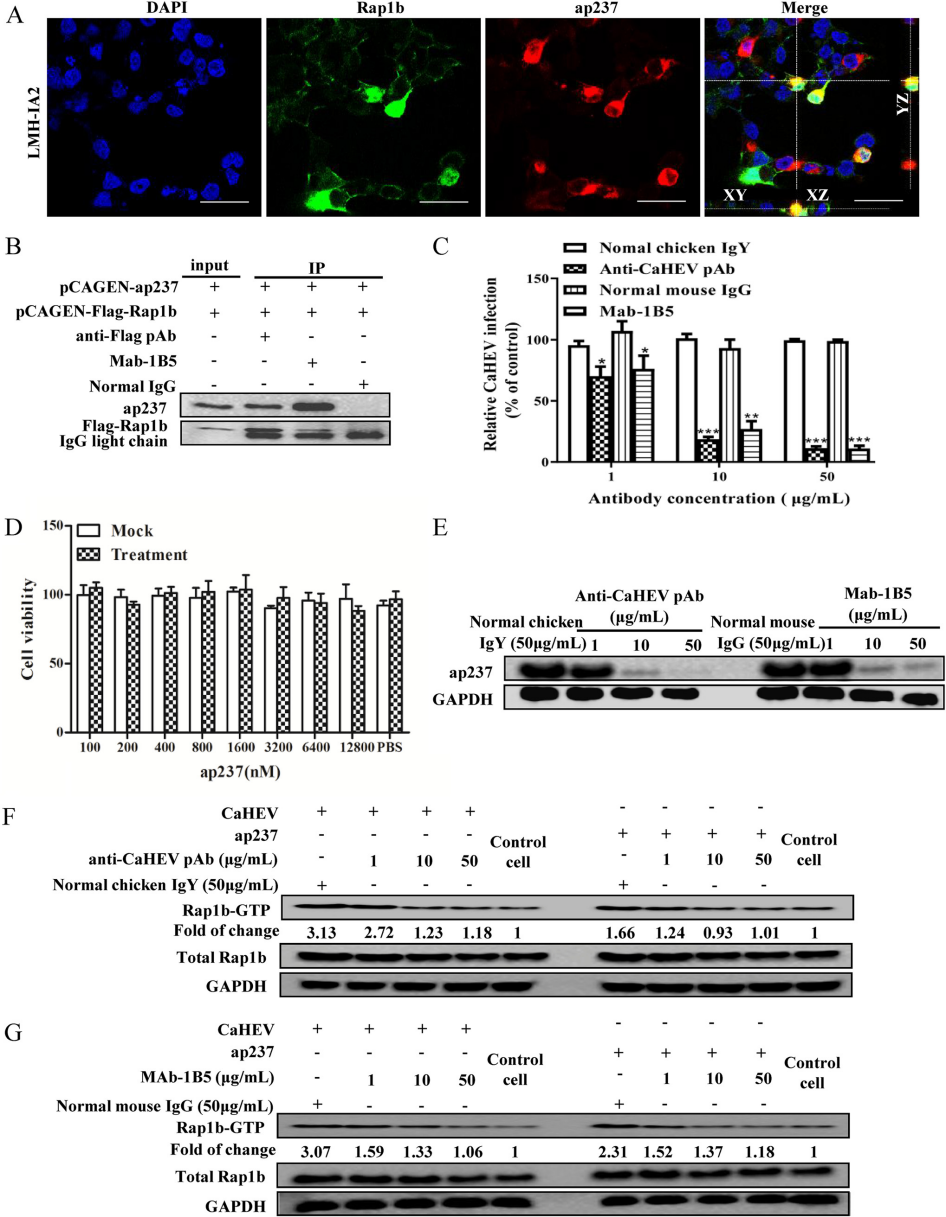
The interaction of Rap1b and the CaHEV capsid is required for Rap1b activation. (A) LMH-IA2 cells were cotransfected with pCAGEN-ap237 and pCAGEN-Flag-Rap1b for 48 h. Next, cells were fixed with 4% paraformaldehyde and permeabilized using PBS containing 0.5% Triton X-100, followed by cell staining using ap237-specific Mab-1B5 (red channel) and anti-Flag rabbit polyclonal antibody (green channel) for confocal microscopy (scale bar, 20 μm). (B) LMH-IA2 cells were transfected with pCAGEN-ap237 and pCAGEN-Flag-Rap1b for 48 h. Next, the cells were harvested using NP40 lysis buffer for the Co-IP assay using ap237-specific Mab-1B5 and anti-Flag rabbit polyclonal antibody (anti-Flag pAb). Cell lysates supplemented with normal mouse IgG (normal IgG) were included as antibody isotype controls. (C) Polyclonal IgY purified from CaHEV-infected chicken sera (anti-CaHEV pAb) and Mab-1B5 were preincubated with CaHEV at different concentrations before incubation of the LMH-1A2 cells at 4°C for 2 h to allow virion attachment. Next, the cells were transferred to 37°C for 1 h to induce virion internalization. After removing noninternalized virions, the internalized virus was assessed by qPCR. Normal chicken IgY and mouse IgG were used as antibody isotype controls. Error bars represent the variation from at least three independent experiments. Statistical significance between the indicated groups is marked as * or **, denoting *P* <0.05 or *P < *0.01. (D) CCK-8 cytotoxicity analysis of LMH-1A2 cells incubated with different doses of ap237 protein. Error bars represent the variation from at least three independent experiments. (E) The anti-CaHEV pAb and Mab-1B5 were mixed and incubated with ap237 protein at different concentrations before treating LMH-1A2 cells at 4°C for 1 h to allow protein attachment. After removing unbound protein, the cells were transferred to 37°C for 1 h to induce internalization, whereas noninternalized ap237 protein was removed with citrate buffer. Internalized ap237 was assessed by Western blotting using Mab-1B5. (F) CaHEV and ap237 proteins were mixed with different doses (1 μg/mL, 10 μg/mL, and 50 μg/mL) of CaHEV-pAb, and then mixtures were added to LMH-1A2 cells for an incubation time of 2 h for a mixture of CaHEV-pAb or 1 h for a mixture of ap237-pAb. After washing the cells with PBS 3 times, the cells were transferred to 37°C for 1 h before being harvested to assess the Rap1b and Rap1b-GTP levels. CaHEV or ap237 protein mixed with 50 μg/mL normal chicken IgY (normal IgY) was included as an antibody isotype control. (G) CaHEV and ap237 proteins were mixed with different doses (1 μg/mL, 10 μg/mL, and 50 μg/mL) of Mab-1b5, and then the mixtures were added to LMH-1A2 cells for an incubation time of 2 h for a mixture of CaHEV-Mab-1B5 or 1 h for a mixture of ap237-Mab-1B5. After washing the cells with PBS 3 times, the cells were transferred to 37°C for 1 h before being harvested to assess the Rap1b and Rap1b-GTP levels. CaHEV or ap237 protein mixed with 50 μg/mL normal mouse IgG was included as an antibody isotype control.

Conversely, to determine whether the interaction of the CaHEV capsid and Rap1b was required to promote Rap1b upregulation and activation, a colocalization assay and Co-IP assay were performed. As demonstrated in Fig. 3A, when ap237 and Rap1b were coexpressed in LMH-1A2 cells, a colocalization pattern could be observed for ap237 and Rap1b. Concurrently, a direct interaction between ap237 and Rap1b was detected using a Co-IP assay (Fig. 3B), which suggests the presence of a direct interaction between Rap1b and the CaHEV capsid. Moreover, to further verify that the enhanced activation of Rap1b was a consequence of the Rap1b-capsid interaction, ORF2 polyclonal IgY purified from CaHEV convalescent chicken sera and ORF2-specific Mab-1B5 were employed to block the attachment of CaHEV or ap237 protein to LMH-1A2 cells, whereby the Rap1b-capsid interaction was concurrently interrupted by antibodies. As demonstrated in Fig. 3C, both CaHEV-specific IgY (polyclonal) and Mab-1B5 effectively blocked the attachment of CaHEV to LMH-1A2 cells in a dose-dependent manner. Moreover, when recombinant ap237 protein was employed as a CaHEV capsid mimic to treat LMH cells, minimum cytotoxicity of the ap237 protein was observed (Fig. 3D), but application of either CaHEV-specific IgY or Mab-1B5 blocked attachment of the ap237 protein to LMH-1A2 cells in a dose-dependent manner as well (Fig. 3E). Next, by disrupting attachment of CaHEV and ap237 protein to LMH-1A2 cells, the activation status of Rap1b was significantly inhibited and correlated with increased doses of antibodies (Fig. 3F and G), whereas control antibodies (normal IgY and mouse IgG) demonstrated minimum effects (Fig. 3F and G). Taken together, based on the above-mentioned data, we propose that a direct interaction between the CaHEV capsid protein and Rap1b triggers Rap1b upregulation and activation during the early stage of CaHEV infection, further implying that Rap1b participates in CaHEV virion internalization into permissive cells.

### Rap1b is required for effective virion internalization during CaHEV infection.

**FIG 4:**
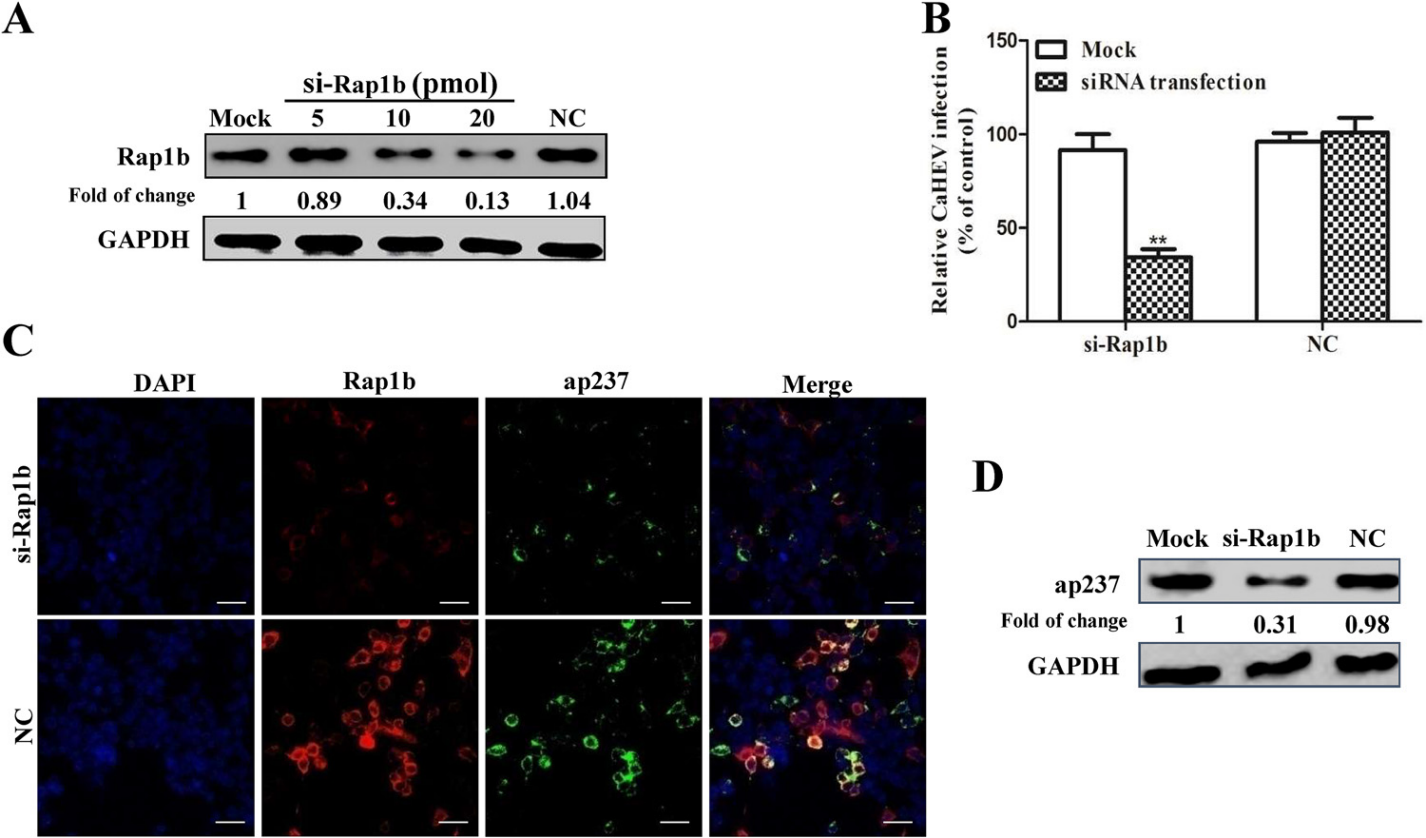
Rap1b is required for effective CaHEV infection. (A) LMH-1A2 cells were transfected with different doses (5, 10, and 20 pmol) of Rap1b-specific siRNA (si-Rap1b) or 20 pmol of control siRNA (NC) for 24 h. Then, the cells were harvested for Western blotting to evaluate Rap1b levels. (B) LMH-1A2 cells were transfected with 20 pmol Rap1b-specific siRNA (si-Rap1b) and control siRNA (NC) for 24 h. Then, the cells were incubated with CaHEV at 4°C for 2 h to allow virion attachment. Next, the cells were transferred to 37°C for 1 h to trigger virion internalization. After removing noninternalized virions, internalized viruses were assessed by qPCR. Cells without siRNA transfection (Mock) were included as controls. Error bars represent the variation from at least three independent experiments. Statistical significance between the indicated groups is marked as **, *P < *0.01. (C) LMH-1A2 cells were transfected with 20 pmol Rap1b-specific siRNA (si-Rap1b) and control siRNA (NC) for 24 h before incubation with ap237 protein at 4°C for 1 h to allow protein attachment, followed by transfer to 37°C for 1 h to trigger internalization. After removing noninternalized proteins, cells were stained for Rap1b (red channel) and ap237 (green channel) in IFA to evaluate the internalization of ap237 protein. (scale bar: 40 μm). (D) LMH-1A2 cells were transfected with 20 pmol Rap1b-specific siRNA (si-Rap1b) and control siRNA (NC) for 24 h, incubated with ap237 protein at 4°C for 1 h, and then transferred to 37°C for 1 h to trigger internalization. After removing noninternalized proteins, cells were harvested for Western blotting to evaluate internalized ap23 protein using Mab-1B5.

To further investigate the role played by Rap1b during CaHEV infection and internalization, siRNA-mediated knockdown was conducted, and a dose-dependent knockdown of Rap1b protein was observed after transfection of LMH-1A2 with Rap1b-specific siRNA (Fig. 4A), whereas negative control siRNA (NC) demonstrated a minimal knockdown effect (Fig. 4A). Next, CaHEV was used to inoculate siRNA-transfected cells, and virion internalization was monitored by qPCR. The results revealed a significant reduction in internalized viral RNA in LM-1A2 cells after knockdown of Rap1b expression (Fig. 4B). Concurrently, subsequent experiments employing ap237 protein as a mimic of CaHEV capsid demonstrated that knockdown of Rap1b expression significantly impaired internalization of ap237 protein by LMH-1A2 cells, as demonstrated by confocal microscopy and Western blotting (Fig. 4C and D), which was consistent with CaHEV infection of LMH-1A2 cells after knockdown of Rap1b. Therefore, these data suggested that Rap1b participated in the internalization of HEV virions, as supported by results showing that knockdown of Rap1b expression impaired HEV internalization by permissive cells.

### Activation of Rap1b contributes to enhanced internalization of CaHEV by permissive cells.

**FIG 5:**
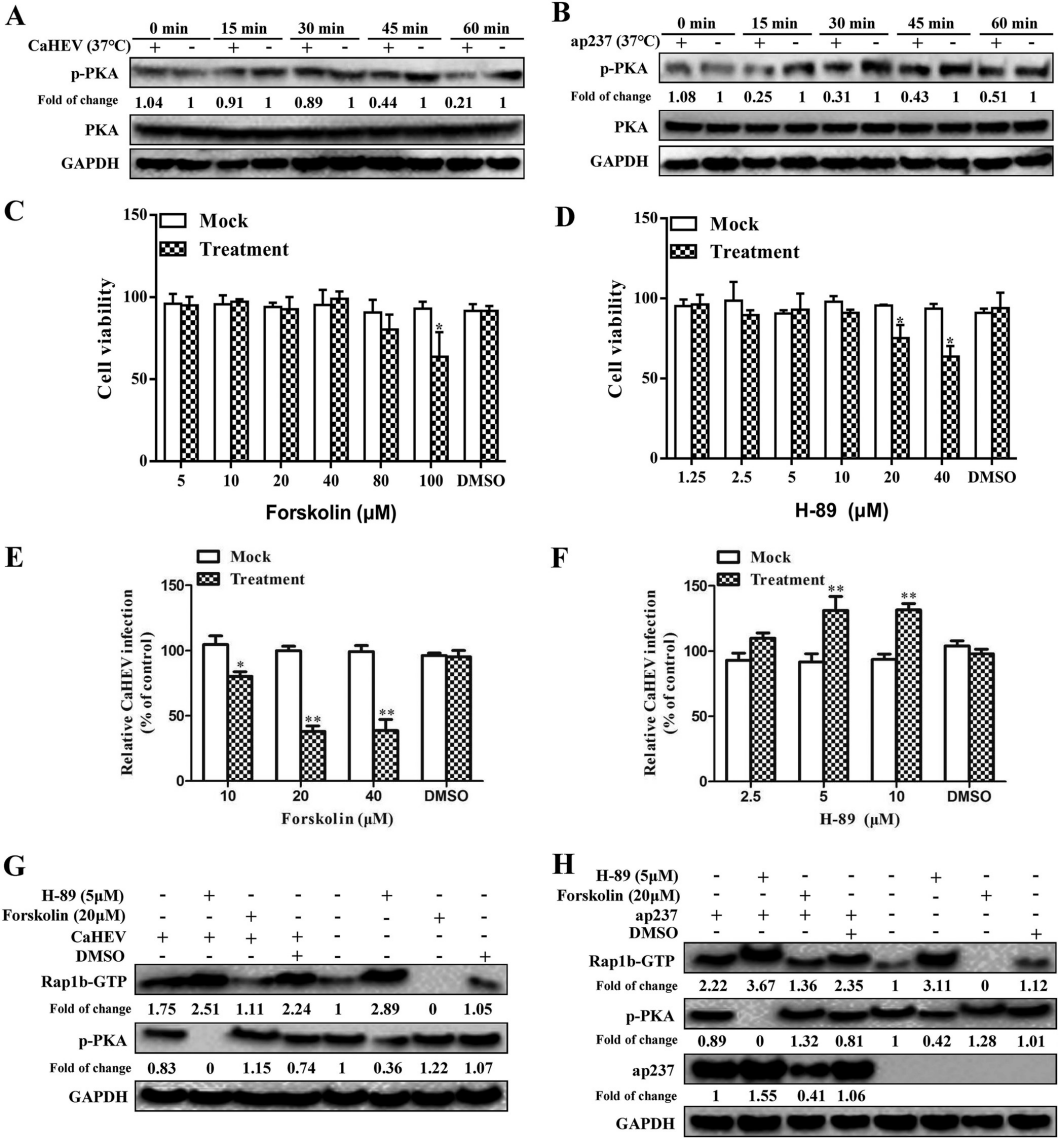
Dephosphorylation of PKA promotes CaHEV-induced activation of Rap1b. (A) LMH-1A2 cells were incubated with CaHEV at 4°C for 2 h and then transferred to 37°C for different times (0, 15, 30, 45, and 60 min) to trigger virion internalization. Next, the cells were harvested for Western blotting to evaluate the phosphorylation of PKA and PKA at global levels. GAPDH served as the protein loading control. (B) LMH-1A2 cells were incubated with ap237 protein at 4°C for 1 h to allow protein attachment and then transferred to 37°C for different times (0, 15, 30, 45, and 60 min) to trigger protein internalization. Next, the cells were harvested for Western blotting to evaluate the phosphorylation of PKA and PKA at the global level. (C) LMH-1A2 cells were treated with the PKA agonist forskolin at different concentrations, and cytotoxicity was assessed using CCK-8 cytotoxicity analysis. (D) LMH-1A2 cells were treated with the PKA antagonist H-89 at different concentrations, and cytotoxicity was assessed using CCK-8 cytotoxicity analysis. (E) LMH-1A2 cells were pretreated with forskolin (0, 20, and 40 μM) for 1 h and then incubated with CaHEV in the presence of forskolin at 4°C for 2 h to allow virion attachment, followed by transfer to 37°C for 1 h to induce virion internalization. After removing noninternalized virions, cells were harvested for detection of internalized virus by qPCR. DMSO-treated cells were included as solvent controls. Error bars represent the variation from at least three independent experiments. Statistical significance between the indicated groups is marked as * or **, denoting *P < *0.05 or *P < *0.01. (F) LMH-1A2 cells were pretreated with H-89 in the same way as described for forskolin above, followed by virion attachment and internalization procedures. Then, the cells were harvested for detection of internalized virus by qPCR. Error bars represent variation from at least three independent experiments. Statistical significance between the indicated groups is marked as **, *P < *0.01. (G) LMH-1A2 cells were pretreated with either 20 μM forskolin or 5 μM H-89 at 37°C for 1 h and then incubated with CaHEV in the presence of drugs at 4°C for 2 h to allow virion attachment followed by transfer to 37°C for 1 h to induce virion internalization. Then, the cells were harvested for Western blotting to evaluate the phosphorylation of PKA and Rap1b-GTP. (H) LMH-1A2 cells were treated with drugs following the same procedure as in Panel G and then incubated with ap237 protein in the presence of drugs at 4°C for 1 h to allow protein attachment followed by transfer to 37°C for 1 h to induce internalization. The phosphorylation of PKA and Rap1b-GTP along with internalized ap237 protein were assessed by Western blotting using the corresponding antibodies.

Membrane-associated small G proteins, such as Rap1b, are normally regulated by PKA-dependent phosphorylation at carboxy-terminal amino acids of Rap1b, whereas PKA-dependent Rap1b phosphorylation inhibits Rap1b activation (25). Thus, the activation status of the upstream regulator PKA (determined as phosphorylated PKA) was evaluated in CaHEV-infected and ap237-inoculated cells. Our results revealed that within 15 min of triggering CaHEV virion internalization by a temperature switch to 37°C, decreased phosphorylation of PKA was observed and maintained at a lower level until 60 min after the temperature switch when compared to cells without CaHEV infection (Fig. 5A). Moreover, in ap237-inoculated cells, there was a rapid decrease in PKA phosphorylation within 15 min of the temperature switch, whereas a partial restoration of PKA phosphorylation could be observed at later time points (30, 45, 60 min) but was still lower than that of cells without ap237 inoculation (Fig. 5B). Thus, a negative correlation between PKA phosphorylation and CaHEV virion internalization was linked. To further confirm these observations, LMH-1A2 cells were pretreated with the PKA agonist forskolin or the PKA antagonist H-89 to induce PKA phosphorylation (PKA activator) or PKA dephosphorylation (PKA inhibitor), respectively. The concentrations of forskolin and H-89 used to treat cells were determined by the cytotoxicity assay (Fig. 5C and D). As demonstrated in Fig. 5E and F, the application of forskolin to evoke PKA activation strongly inhibited CaHEV internalization, as reflected by reduced viral RNA levels in forskolin-treated cells (Fig. 5E), whereas treatment of the same cells with the PKA antagonist H-89 promoted virion internalization (Fig. 5F). Moreover, in alignment with the regulatory role of PKA on Rap1b activity, CaHEV-induced activation of Rap1b was inhibited by treatment with the PKA agonist forskolin but was promoted by treatment with the antagonist H89 (Fig. 5G). Accordingly, uptake of ap237 protein by LMH-1A2 cells was inhibited by forskolin but promoted by the antagonist H89 (Fig. 5H). Taken together, these data suggested that Rap1b activation status controlled CaHEV virion internalization, since functionally blocking or activating upstream regulators of Rap1b could either enhance or inhibit CaHEV internalization by permissive cells, respectively.

### The interaction between Rap1b and the chaperone protein SmgGDS is enhanced during CaHEV internalization.

**FIG 6:**
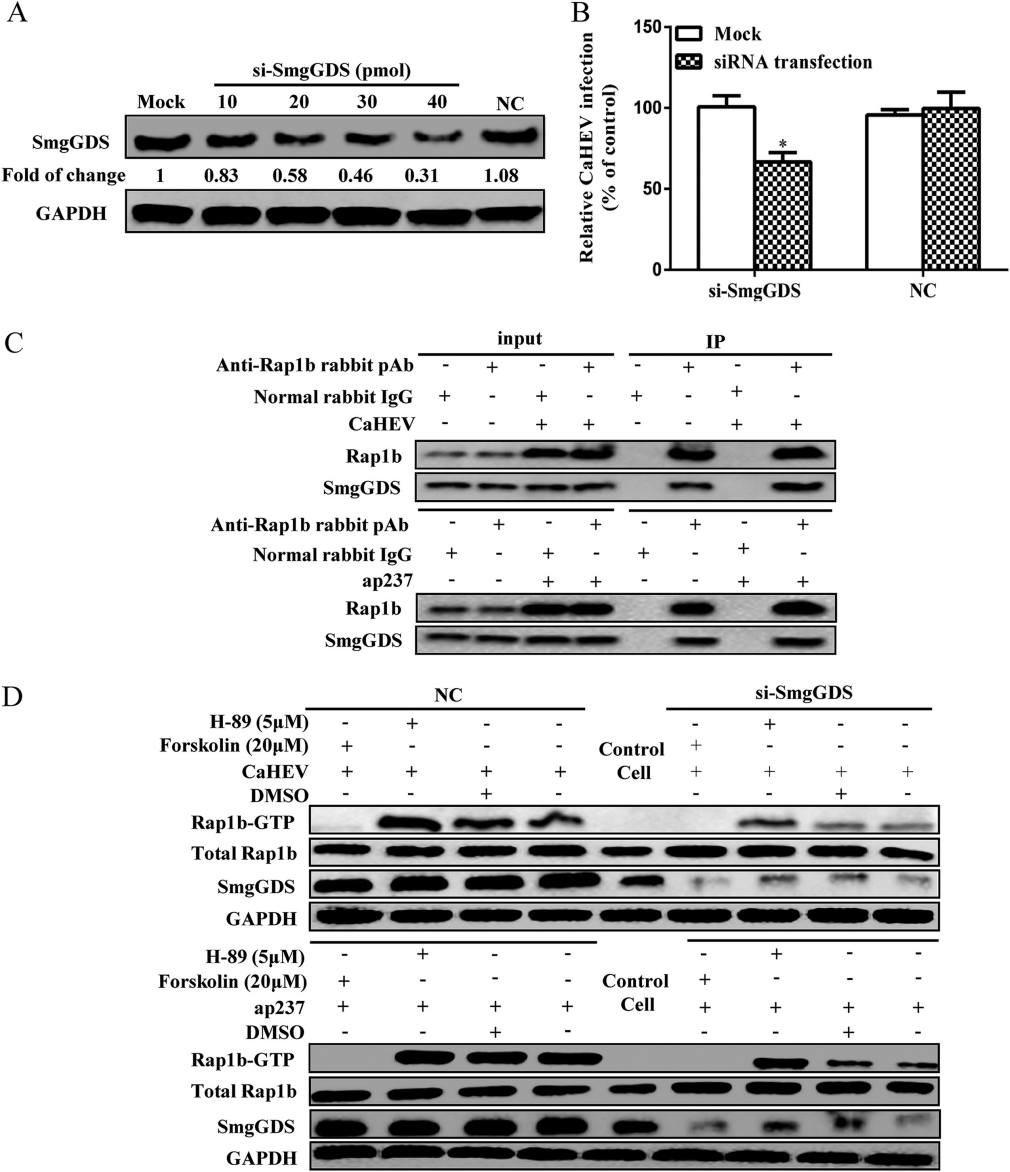
The chaperone protein SmgGDS is essential for CaHEV-induced Rap1b activation. (A) LMH-1A2 cells were transfected with SmgGDS-specific siRNA (si-SmgGDS, 10, 20, 30, 40 pmol) along with control siRNA (NC, 40 pmol) for 24 h, and then the cells were harvested to evaluate SmgGDS protein by Western blotting. (B) LMH-1A2 cells were transfected with 40 pmol of si-SmgGDS and NC siRNAs for 24 h, and then the cells were incubated with CaHEV at 4°C for 2 h of virion attachment followed by transfer to 37°C for 1 h to induce virion internalization. After removing noninternalized virions, the internalized virus was evaluated by qPCR. Error bars represent variation from at least three independent experiments. Statistical significance between the indicated groups is marked as *, *P < *0.05. (C) LMH-1A2 cells were incubated with CaHEV or ap237 protein for attachment and internalization stimulation, and then the cells were harvested for Co-IP assay using anti-Rap1b pAb as bait antibody to detect the interaction between Rap1b and SmgGDS. Normal rabbit IgG was included as an antibody isotype control. (D) LMH-1A2 cells were transfected with SmgGDS-specific siRNA (si-SmgGDS 40 pmol) along with control siRNA (NC, 40 pmol) for 24 h, followed by treatment with either forskolin or H-89 at 37°C for 1 h before incubation with CaHEV or ap237 protein in the presence of drugs at 4°C for 2 h (for CaHEV) or 1 h (for ap237) to allow virion/protein attachment. After transfer to 37°C for 1 h to induce virion/protein internalization, the cells were harvested to evaluate total Rap1b and Rap1b-GTP by Western blotting.

The cell distribution of Rap1b relies on the prenylation- or phosphorylation-dependent regulation of the CAAX box within the C-terminal region of the Rap1b protein. SmgGDS, a chaperone protein, binds to the polybasic region (PBR) site of prenylated Rap1b located upstream of the CAAX box. Next, GEF activity promotes the replacement of Rap1b GDP with GTP (26). The interaction between SmgGDS and Rap1b can be disrupted by activated PKA, thus preventing subsequent prenylation, membrane redistribution, and final activation (GTP loading) of Rap1b (25). Thus, it will be important to elucidate the role played by SmgGDS during CaHEV internalization. To achieve this goal, we first used SmgGDS-specific siRNA to knock down SmgGD protein (Fig. 6A), and we found that downregulation of SmgGDS expression significantly impaired CaHEV internalization (Fig. 6B). Conversely, to further confirm the involvement of SmgGDS in CaHEV infection, the interaction between Rap1b and SmgGDS was investigated using Co-IP, our results demonstrated that the interaction between Rap1b and SmgGDS could be detected in LMH-1A2 cells using Co-IP regardless of CaHEV infection or ap237 protein inoculation (Fig. 6C).

Moreover, the activation of Rap1b was examined in SmgGDS siRNA-transfected cells pretreated with PKA agonist or antagonist, followed by CaHEV infection or ap237 inoculation. Based on our results in CaHEV-inoculated LMH-1A2 and ap237-incubated LMH-1A2 cells, the knockdown of SmgGDS expression led to significantly reduced Rap1b-GTP levels, while total Rap1b levels were similar across the experimental groups (Fig. 6D). Notably, addition of the PKA antagonist H-89 led to a partial restoration of Rap1b activation status (Fig. 6D), which was accompanied by a slight increase in total SmgGDS levels in both CaHEV-infected and ap237-incubated LMH-1A2 cells compared with normal cells (Fig. 6D). Therefore, these data suggested that SmgGDS was involved in enhanced activation of Rap1b during the interaction between capsid and Rap1b and thereby required for CaHEV internalization.

### Internalization of CaHEV promotes membrane redistribution of RIAM.

**FIG 7:**
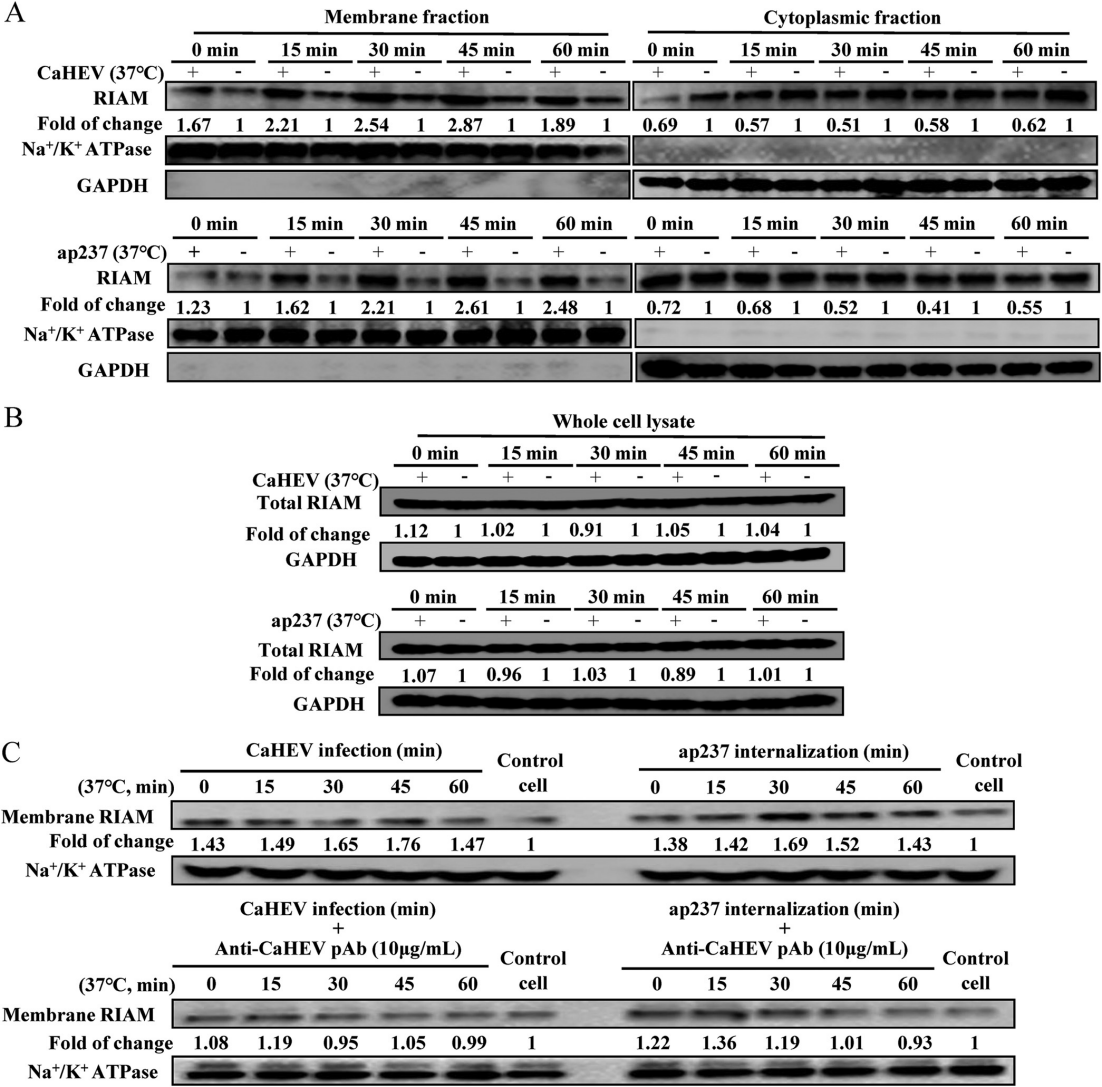
Membrane redistribution of RIAM is involved in CaHEV infection. (A) LMH-1A2 cells were incubated with either CaHEV or ap237 protein at 4°C for 2 h or 1 h for virion or protein attachment, respectively. Next, the cells were transferred to 37°C for different times (0, 15, 30, 45. and 60 min) to induce internalization. The membrane (left panel) and cytoplasmic (right panel) fractions were separated to monitor dynamic changes in the membrane distribution of RIAM. Na^+^/K^+^ ATPase and GAPDH were used to confirm the separation of membrane and cytoplasm fractions. (B) Whole-cell lysates of samples with the same treatment as Panel A were harvested to evaluate global RIAM expression. (C) Purified CaHEV-specific IgY (anti-CaHEV pAb) was premixed with CaHEV or ap237 protein, and then LMH-1A2 cells were incubated with both mixtures at 4°C for 2 h (CaHEV-pAb mixture) or 1 h (ap237-pAb mixture) for attachment. Next, the cells were transferred to 37°C for different times (0, 15, 30, 45, and 60 min) to induce internalization. The membrane fractions were harvested to monitor changes in the membrane distribution of RIAM. Cells inoculated with CaHEV and ap237 protein without mixing of antibody but harvested at the same times were included as controls (upper panel).

RIAM, the downstream effector of Rap1b, has been shown to participate in inside-out integrin signaling depending on its membrane recruitment induced by activated Rap1b (27). To determine whether RIAM participated in CaHEV internalization-induced Rap1b activation, CaHEV-infected or ap237-incubated LMH-1A2 cells were subjected to membrane-cytoplasmic subfractionation to monitor the subcellular distribution of RIAM at different time points after the temperature switch from 4°C to 37°C. Our results revealed that membrane redistribution of RIAM occurred after virion attachment to cells even in the absence of a temperature switch (0 min), with enhanced membrane redistribution of RIAM lasting for 60 min after temperature switch-based triggering of virion or ap237 internalization (Fig. 7A). Consistent with the increase in RIAM protein levels in the membrane fraction after the temperature switch, a corresponding decrease in RIAM levels was observed in the cytoplasmic fraction of CaHEV-infected or ap237-incubated LMH-1A2 cells (Fig. 7A). Concurrently, total RIAM expression from the whole cell lysate was unaltered in either CaHEV-infected or ap237-inoculated LMH-1A2 cells at all time points examined (Fig. 7B), thus further indicating that increased membrane recruitment of RIAM in CaHEV-infected or ap237-inoculated cells was a direct consequence of internalization of virions or viral capsids rather than altered expression of RIAM.

In contrast, to further verify that CaHEV-induced membrane recruitment of RIAM required cell membrane attachment of the CaHEV virion or capsid, CaHEV-specific polyclonal IgY (pAb) and Mab-1B5 were employed to block attachment of the CaHEV virion or ap237 to LMH-1A2 cells. As demonstrated in Fig. 7C, the blockade of membrane attachment by CaHEV or ap237 protein prevented the membrane redistribution of RIAM at all time points examined after the temperature switch to 37°C (Fig. 7C) compared with cells incubated with CaHEV or ap237 protein alone. Therefore, the above-mentioned results suggested that membrane recruitment of RIAM within permissive cells was a consequence of Rap1b activation triggered by CaHEV or ap237 attachment and internalization.

### CaHEV internalization-induced membrane redistribution of RIAM promotes membrane anchoring of Talin-1.

**FIG 8:**
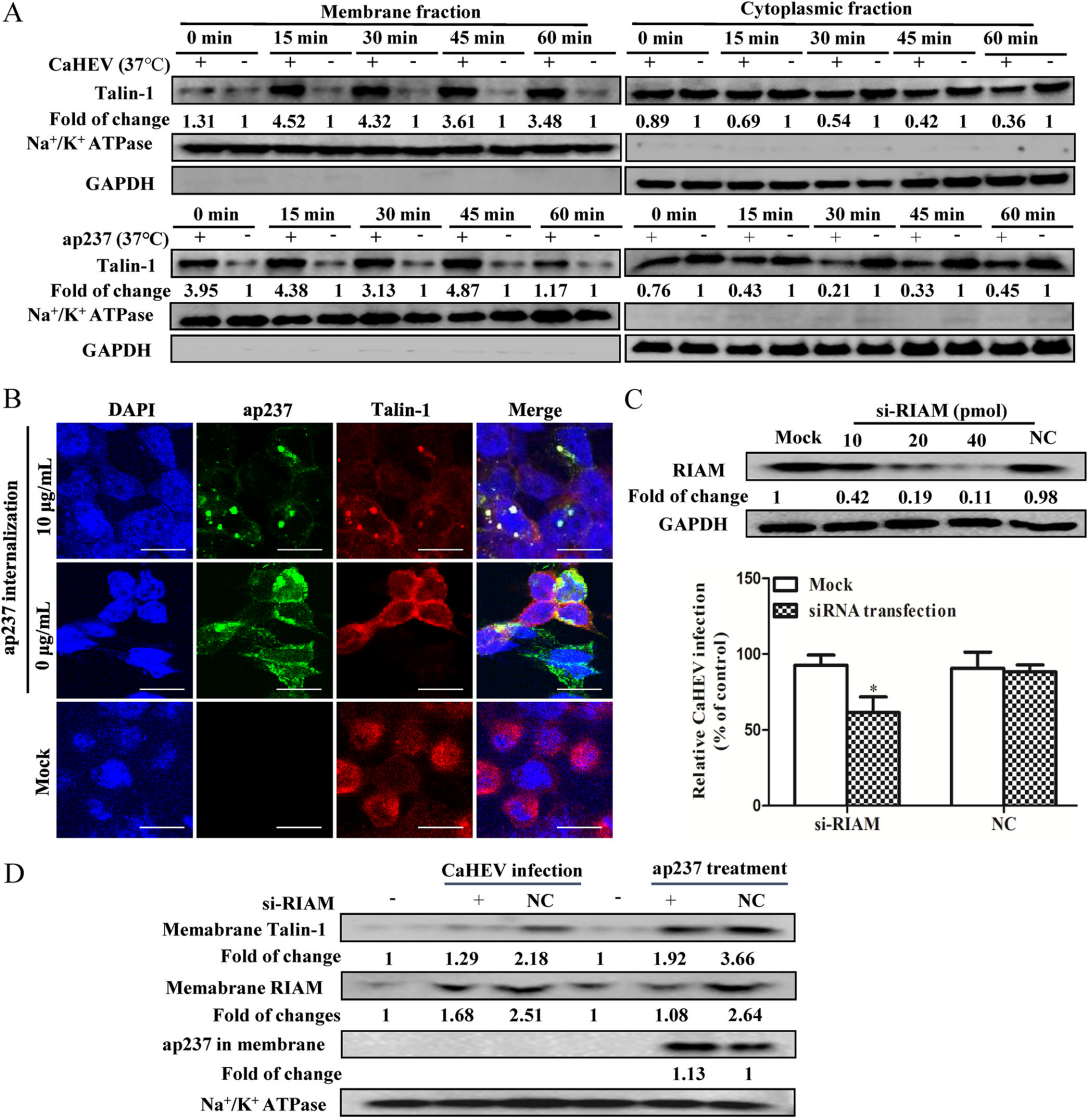
Rap1b-RIAM signaling promotes the membrane anchoring of Talin-1 during CaHEV infection. (A) LMH-1A2 cells were incubated with CaHEV or ap237 protein at 4°C for attachment. Then, the cells were transferred to 37°C for different times to trigger internalization. The membrane (left panel) and cytoplasmic (right panel) fractions were separated and harvested for Western blotting to evaluate Talin-1 protein levels in both fractions. Na^+^/K^+^ ATPase and GAPDH were evaluated to confirm the separation of membrane and cytoplasm fractions. (B) LMH-1A2 cells were incubated with a mixture of purified anti-CaHEV pAb and ap237 protein at 4°C for 1 h and then transferred to 37°C for 1 h. Then, the distribution of Talin-1 and capsid protein was stained using an immunofluorescence assay. (C) LMH-1A2 cells were transfected with RIAM-specific siRNA (si-RIAM, 10, 20, and 40 pmol) or control siRNA (NC, 40 pmol) for 24 h, followed by the evaluation of RIAM protein by Western blotting (upper panel). Next, cells transfected with si-RIAM and NC (40 pmol) siRNAs were incubated with CaHEV at 4°C for 2 h and then transferred to 37°C for 1 h to induce internalization. After removing the noninternalized virion, the internalized virus was evaluated using qPCR. Error bars represent the variation from at least three independent experiments. Statistical significance between the indicated groups is marked as *, *P < *0.05. (D) LMH-1A2 cells were transfected with siRNAs as described in Panel C and then incubated with either CaHEV or ap237 protein at 4°C for 2 h or 1 h for attachment. Next, the cells were transferred to 37°C for 1 h to induce internalization. The membrane fraction was extracted to evaluate the distribution of Talin-1 and noninternalized ap237 protein by Western blotting.

The N-terminus of RIAM binds Talin-1, which is a cytoskeletal protein known to interact with cytoplasmic tails of integrins to regulate their cell-surface adhesive states (28, 29). Normally, the inactive form of Talin-1 exhibits a random cell distribution due to intramolecular self-binding that inhibits Talin-1-associated intermolecular interactions (29). During the membrane recruitment process of Talin-1 induced by membrane redistribution of RIAM, interaction occurs between membrane-anchored Talin-1 and integrin cytoplasmic tails, thereby subsequently generating inside-out integrin signaling (22, 30–32). To determine whether these cascades occurred during CaHEV infection, we investigated whether Talin-1 was recruited to the cell membrane in a manner mirroring membrane recruitment of RIAM during CaHEV infection. As demonstrated via a membrane-cytoplasmic subcellular fractionation assay, induction of CaHEV or ap237 internalization by a temperature switch simultaneously triggered Talin-1 membrane recruitment at all examined time points (Fig. 8A, left panel). Moreover, reduced Talin-1 levels were observed in the corresponding cytoplasmic fractions (Fig. 8A, right panel), which were inversely proportional in magnitude to increases in Talin-1 membrane recruitment. In addition, confocal microscopy-based observations indicated that blockade of ap237 attachment to LMH-1A2 cells by polyclonal antibodies led to a reduction in membrane-anchored Talin-1 (Fig. 8B). Thus, membrane anchoring of Talin-1 participated in CaHEV attachment and internalization during CaHEV infection.

To uncover the exact roles played by RIAM and Talin-1 during CaHEV internalization, siRNA was applied to knock down the RIAM protein. Consequently, RIAM knockdown significantly impaired CaHEV internalization by LMH-1A2 cells (Fig. 8C). Moreover, RIAM knockdown also led to reduced internalization of the ap237 protein (as evidenced by increased retention of ap237 in the membrane fraction), along with reduced Talin-1 membrane redistribution (Fig. 8D). Taken together, these results indicated that RIAM and Talin-1, adaptors downstream of Rap1b, were redistributed to the cytoplasmic membrane during CaHEV infection, whereas RIAM and Talin-1 were not involved in virion attachment but participated in virion internalization.

### CaHEV-induced membrane-anchored Talin-1 is associated with integrin α5/β1 activation.

**FIG 9:**
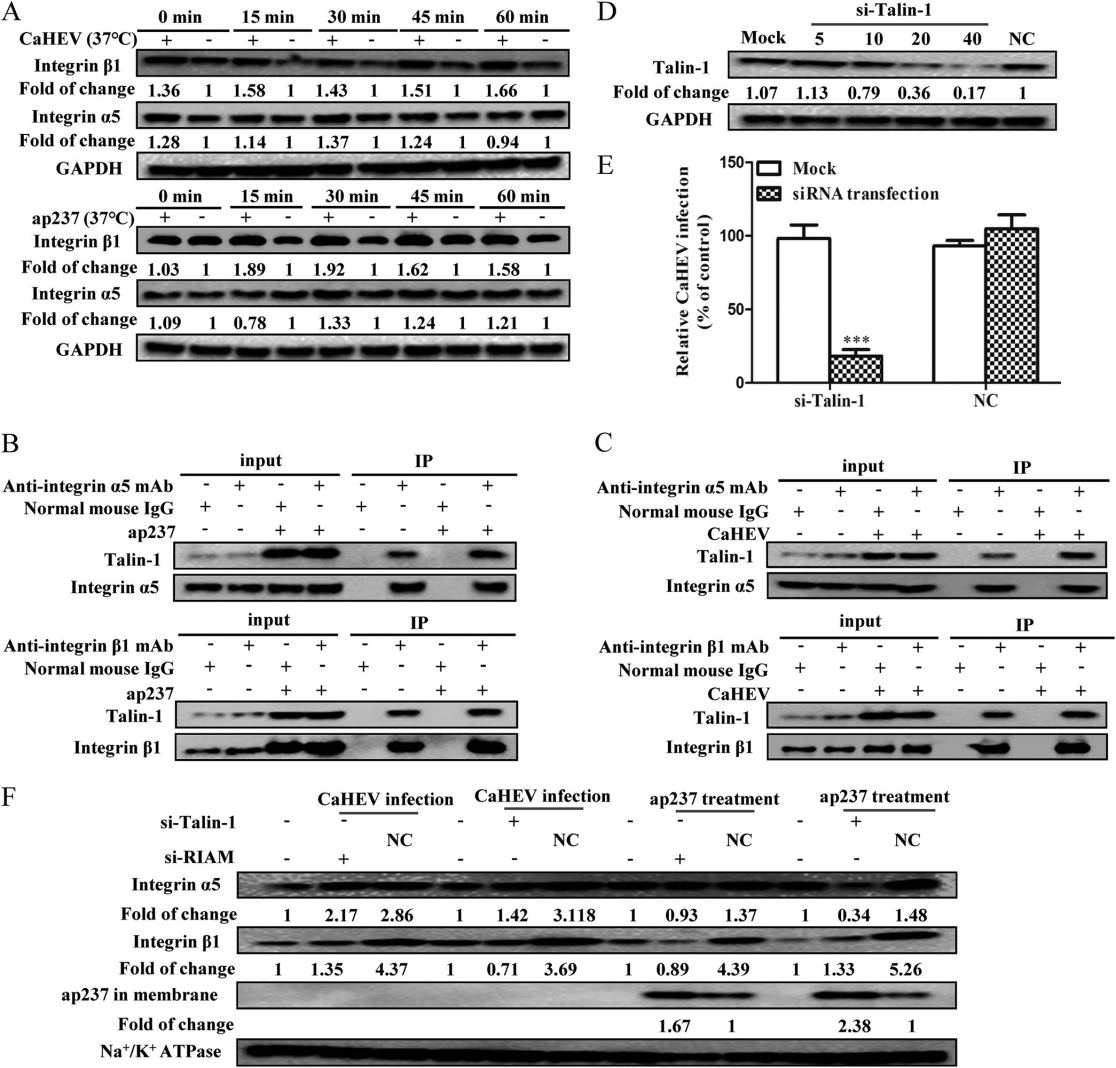
The RIAM-Talin-1 signal cascade activates integrin α5/β1. (A) LMH-1A2 cells were incubated with either CaHEV or ap237 protein at 4°C for 2 h or 1 h for attachment, respectively. Then, the cells were transferred to 37°C for different times (0, 15, 30, 45, and 60 min) to induce internalization. Next, the cells were harvested for Western blotting to evaluate integrin α5 and integrin β1. (B) LMH-1A2 cells were incubated with ap237 at 4°C for 1 h and then transferred to 37°C for 1 h for protein internalization. Next, the cells were harvested, and the interaction between Talin-1 and integrin α5 or between Talin-1 and integrin β1 was evaluated by coimmunoprecipitation. (C) LMH-1A2 cells were incubated with CaHEV at 4°C for 2 h and then transferred to 37°C for 1 h to induce internalization. Next, the cells were harvested, and the interaction between Talin-1 and integrin α5 or between Talin-1 and integrin β1 was evaluated by coimmunoprecipitation. (D) LMH-1A2 cells were transfected with Talin-1-specific siRNA (si-Talin-1, 5, 10, 20, and 40 pmol) and control siRNA (NC, 40 pmol) for 24 h. Next, Talin-1 protein levels in siRNA-transfected cells were detected by Western blotting. (E) LMH-1A2 cells were transfected with siTalin-1 and NC siRNAs (40 pmol) for 24 h and then incubated with CaHEV at 4°C for 2 h, followed by transfer to 37°C for 1 h to induce virion internalization. After removing noninternalized virions, the internalized virus was assessed by qPCR. Error bars represent the variation from at least three independent experiments. Statistical significance between the indicated groups is marked as ***, *P < *0.001. (F) LMH-1A2 cells were transfected with 40 pmol si-RIAM or si-Talin-1 along with NC siRNAs for 24 h. Next, the cells were incubated with either CaHEV or ap237 protein at 4°C for attachment before transfer to 37°C for internalization. The membrane fraction was extracted to evaluate the integrin α5, integrin β1, and uninternalized ap237 proteins.

Integrins, a transmembrane receptor family, are heterodimers composed of various α and β chains (18 α, 8 β) that play multiple roles in cell adhesion, migration, and cytoskeleton rearrangement (33). Accumulating evidence indicates that viruses exploit integrin-induced cytoskeleton functions to infect host cells, and integrin α5/β1 has been shown to participate in the early phase of viral infection, specifically during virion binding, fusion, and internalization steps (34–37). Since the interaction between membrane-anchored Talin-1 and integrin cytoplasmic tails confers inactive integrins into a high-affinity activated state (29, 38), we assessed the levels of integrin α5 and β1 in either CaHEV-infected or ap237-incubated LMH-1A2 cells, and a significant upregulation of integrin α5/β1 could be observed at 15 min, 30 min, and 45 min after the temperature switch to 37°C (Fig. 9A), whereas the evaluation of integrin β1 appeared to be higher than that of α5 (Fig. 9A).

To further confirm integrin α5/β1 involvement in CaHEV infection as a consequence of CaHEV-induced activation of the Rap1b-RIAM-Talin-1 axis, the potential interaction between integrin α5/β1 and Talin-1 was examined via Co-IP assays in LMH-1A2 cells after CaHEV attachment and internalization. As expected, interactions between Talin-1 and integrin α5/β1 were observed in both ap237-incubated cells (Fig. 9B) and CaHEV-inoculated cells (Fig. 9C). Moreover, mirroring the scenario of RIAM, knockdown of Talin-1 expression using siRNA significantly impaired CaHEV internalization by LMH-1A2 cells (Fig. 9D and E). Moreover, the application of either RIAM-specific or Talin-1-specific siRNAs to knock down the corresponding targets blocked internalization of the ap237 protein, as reflected by increased retention of the ap237 protein in the membrane fraction of ap237-inoculated LMH-1A2 cells (Fig. 9F). Taken together, these data suggested that Talin-1 was recruited to the cell membrane during CaHEV infection and bound to cytoplasmic tails of integrin α5/β1 to trigger integrin α5/β1 activation as a downstream event after activation of the Rap1b-RIAM-Talin-1 axis by CaHEV attachment and internalization in permissive cells.

### Talin-1-induced integrin α5/β1 activation stimulates FAK-RAC1&CDC42-PAK1-LIMK1-Cofilin signaling during CaHEV infection.

**FIG 10:**
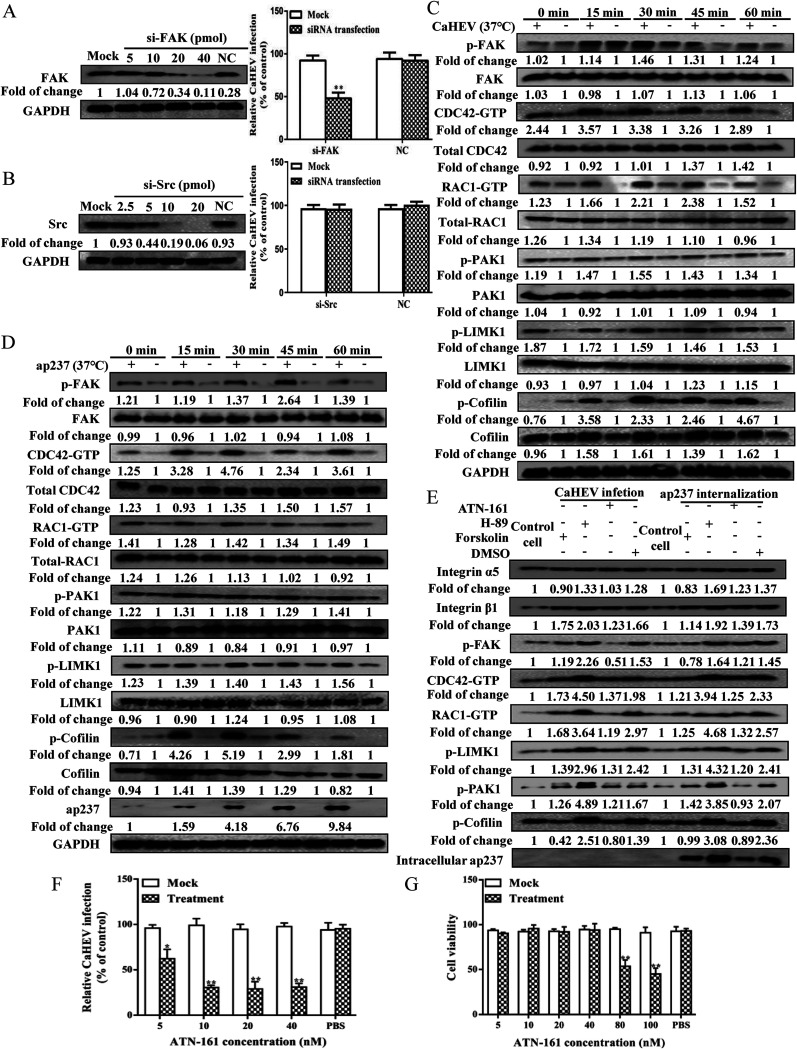
CaHEV infection and viral capsid protein internalization induce activation of the FAK-RAC1&CDC42-PAK1-LIMK1-Cofilin signaling pathway. (A) LMH-1A2 cells were transfected with different concentrations of FAK-specific siRNA (si-FAK, 5, 10, 20, and 40 pmol) and control siRNA (NC, 40 pmol) for 24 h, and the FAK protein levels were assessed by Western blotting (upper panel). Next, cells transfected with siFAK and NC siRNAs (40 pmol) were incubated with CaHEV at 4°C for 2 h followed by transfer to 37°C for 1 h. After removing the noninternalized virion, the internalized virus was detected using qPCR (lower panel). Error bars represent variation from at least three independent experiments. Statistical significance between the indicated groups is marked as **, *P < *0.01. (B) LMH-1A2 cells were transfected with different concentrations of Src-specific siRNA (si-Src, 2.5, 5, 10, 20 pmol) or control siRNA (NC, 20 pmol) for 24 h, and then the Src protein levels were assessed by Western blotting (upper panel). Next, the cells transfected with 10 pmol si-Src and NC siRNAs were incubated with CaHEV at 4°C for 2 h followed by transfer to 37°C for 1 h. After removing the noninternalized virion, the internalized virus was detected using qPCR (lower panel). Error bars represent the variation from at least three independent experiments. (C) LMH-1A2 cells were incubated with CaHEV at 4°C for 2 h and then transferred to 37°C for different times (0, 15, 30, 45, and 60 min) to induce virion internalization. Next, the cells were harvested for analysis of the activation status of downstream molecules in the FAK-RAC1&CDC42-PAK1-LIMK1-Cofilin signaling cascade using the corresponding antibodies by Western blotting. (D) LMH-1A2 cells were incubated with ap237 protein at 4°C for 1 h and then transferred to 37°C for different times (0, 15, 30, 45, and 60 min) to induce internalization. Next, cells were harvested for analysis of the activation status of downstream molecules in the FAK-RAC1&CDC42-PAK1-LIMK1-Cofilin signaling cascade using the corresponding antibodies in Western blotting. (E) LMH-1A2 cells were pretreated with forskolin, H-89, or ATN-161 at 37°C for 1 h and then incubated with either CaHEV or ap237 protein in the presence of drugs at 4°C for 2 h (for CaHEV) or 1 h (for ap237 protein) to allow virion/protein attachment, followed by transfer of the cells to 37°C for 1 h to induce internalization. Next, the cells were harvested, and integrin α5, integrin β1, p-FAK, CDC42-GTP, RAC1-GTP, p-LIMK1, p-PAK1, p-Cofilin, and internalized ap237 protein (intracellular ap237) were examined by Western blotting. (F) LMH-1A2 cells were treated with ATN-161 at different concentrations, and then a CCK-8 cytotoxicity assay was conducted to evaluate the cell viability of ATN-161-treated cells. (G) LMH-1A2 cells were pretreated with ATN-161 at 37°C for 1 h and then incubated with CaHEV in the presence of drug at 4°C for 2 h followed by transfer to 37°C for 1 h to induce internalization. After removing noninternalized virions, the cells were harvested for detection of internalized virus by qPCR. PBS-treated cells were included as a nontreatment control. Error bars represent the variation from at least three independent experiments. Statistical significance between the indicated groups was marked as * or **, denoting *P < *0.05 or *P < *0.01.

Focal adhesion kinase (FAK), which is a nonreceptor tyrosine protein kinase, plays a key role in integrin-mediated signaling (39). FAK is primarily recruited to integrin clustering sites via interactions between the C-terminal domain of FAK and Talin-1, whereby integrin-stimulated phosphorylation of FAK in Y397 creates a high-affinity binding site for SH2 domains of SFKs (e.g., Src). Concurrently, the binding of Src to FAK induces the formation of a transient FAK-Src signaling complex, thus activating FAK/Src-associated downstream signals (40, 41). Therefore, siRNA knockdown of FAK and Src was carried out to evaluate their roles in CaHEV internalization, and the data demonstrated that knockdown of FAK expression significantly inhibited CaHEV internalization, whereas knockdown of Src exerted a minimal effect (Fig. 10A and B), which suggests that FAK-mediated signaling was required for CaHEV internalization.

In addition to FAK, investigation of the downstream signaling molecules RAC1, CDC42, PAK1, LIMK1, and Cofilin during CaHEV internalization indicated that signaling cascades of FAK were strongly activated in CaHEV-infected and ap237-incubated LMH-1A2 cells after an endocytosis-inducing temperature switch (Fig. 10C and D). Specifically, peak activation occurred between 15 and 30 min after switching, as evidenced by increased levels of CDC42-GTP and RAC1-GTP, as well as increased levels of phosphorylated FAK, PAK1 LIMK1, and Cofilin (Fig. 10C and D). Moreover, the PKA agonist forskolin/antagonist H-89 and integrin α5/β1 antagonist ATN-161 were employed to confirm that enhanced activation of FAK and downstream pathways were consequences of Rap1b and integrin α5/β1 activation. As demonstrated in Fig. 10E, in either CaHEV-infected or ap237-inoculated cells, the blockade of Rap1b activation using the PKA agonist forskolin led to impaired activation of corresponding molecules downstream of FAK activation cascades and reduced ap237 internalization, as reflected by reduced intracellular ap237 levels. Conversely, the application of the PKA antagonist H-89 (to activate Rap1b) produced results opposite to those obtained using forskolin (Fig. 10E). Notably, the application of the integrin α5/β1 antagonist ATN-161 to block integrin activation led to impaired activation of corresponding molecules downstream of FAK activation cascades and reduced ap237 internalization (Fig. 10E). Concurrently, application of ATN-161 at a tolerable level of cytotoxicity (Fig. 10G) inhibited virion internalization by CaHEV-infected LMH-1A2 cells (Fig. 10F). Thus, these data suggested that CaHEV attachment and internalization activated both Rap1b and integrin α5/β1 to further trigger FAK-RAC1&CDC42-PAK1-LIMK1-Cofilin signaling and subsequently promote virion internalization.

### CaHEV infection exploits cell membrane remodeling processes.

**FIG 11:**
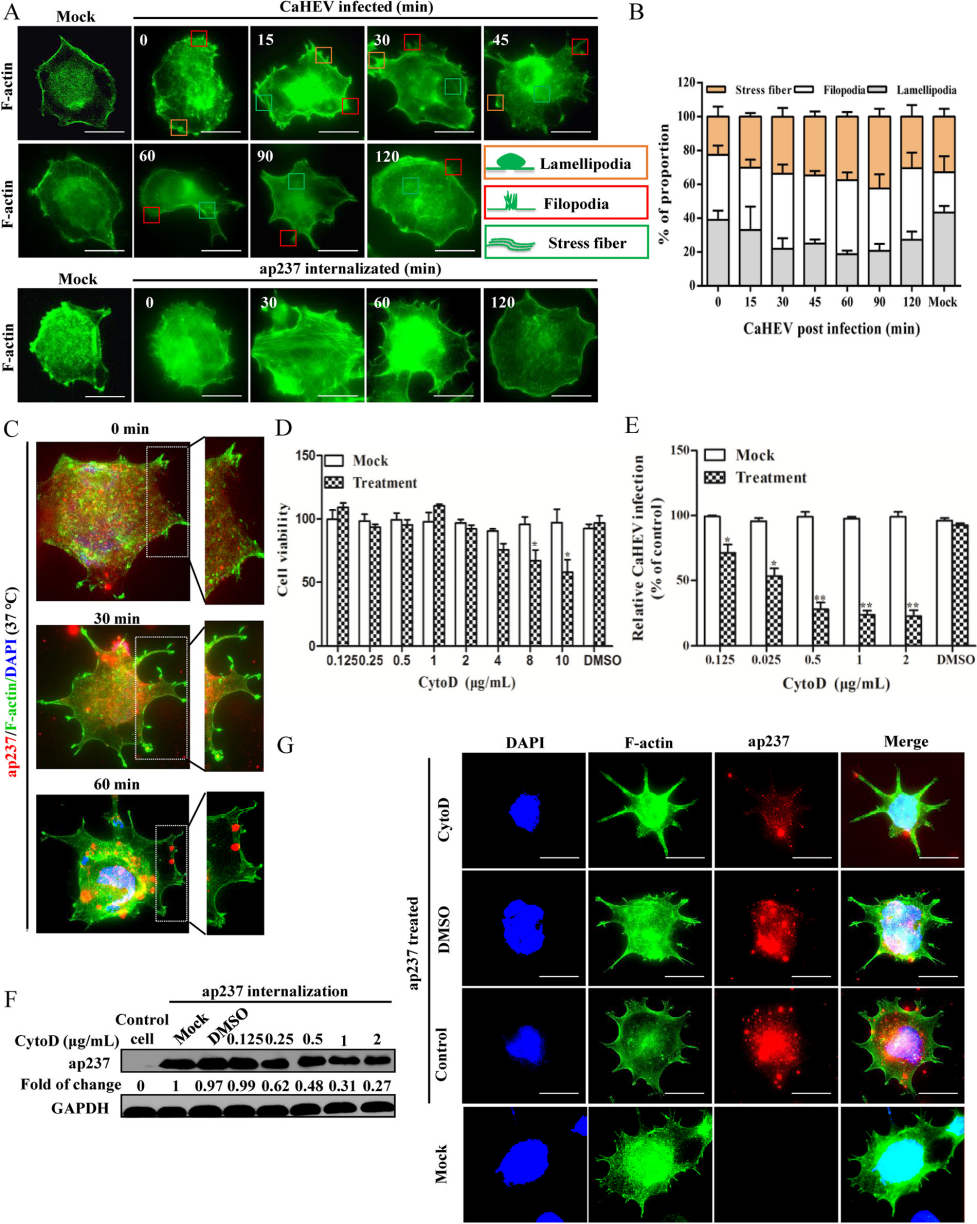
Membrane remodeling induced by the Rap1b-Talin-1-Integrin pathway facilitates CaHEV infection. (A) LMH-1A2 cells were incubated with CaHEV or ap237 protein at 4°C for 2 h (CaHEV) or 1 h (ap237 protein) to allow virion/protein attachment. Then, the cells were transferred to 37°C for different times to induce virion/protein internalization. F-actin was visualized using FITC-labeled phalloidin. (B) LMH-1A2 cells were incubated with CaHEV at 4°C for 2 h to allow virion attachment, followed by transferring cells to 37°C for different times as indicated (15 to 120 min). After visualization of F-actin using FITC-labeled phalloidin, cells with stress fibers, lamellipodia, or filopodia were designated positive cells. The average value of 50 positive cells randomly selected from at least 3 represented regions of 3 separated phalloidin staining experiments was used for quantification. (C) LMH-1A2 cells were incubated with ap237 protein at 4°C for 1 h and then transferred to 37°C for 1 h to induce internalization, followed by staining with FITC-labeled phalloidin (green channel) and Mab-1B5 (red channel) at different time points (0, 30, and 60 min). (D) The F-actin inhibitor CytoD was used to treat LMH-1A2 cells at different concentrations for 24 h, and the cytotoxicity of CytoD was evaluated using a CCK-8 cytotoxicity assay. *, *P < *0.05. (E) LMH-1A2 cells were pretreated with CytoD at different concentrations at 37°C for 1 h and incubated with CaHEV in the presence of CytoD at 4°C for 2 h. Then, the cells were transferred to 37°C for 1 h to induce virion internalization. After removing noninternalized virions, the internalized virus was analyzed by qPCR. Error bars represent the variation from at least three independent experiments. Statistical significance between the indicated groups was marked as * or **, denoting *P < *0.05 or *P < *0.01, respectively. (F) LMH-1A2 cells were pretreated with CytoD at different concentrations at 37°C for 1 h and then incubated with ap237 protein in the presence of drug at 4°C for 1 h. Next, the cells were transferred to 37°C for 1 h to induce internalization. After removing noninternalized ap237, the internalized protein was analyzed by Western blotting. (G) LMH-1A2 cells were pretreated with 1 μg/mL CytoD at 37°C for 1 h and then incubated with ap237 protein in the presence of CytoD at 4°C for 1 h. Next, the cells were transferred to 37°C for 1 h to induce protein internalization before fixation for immunofluorescence assays. The internalized ap237 protein and F-actin were visualized using the corresponding antibody and FITC-labeled phalloidin.

The cellular actin cytoskeleton is regulated by Cofilin, which controls the polymerization and depolymerization of actin filaments associated with actin kinetics (42). The conversion of actin from a monomeric form (G-actin) to a filamentous form (F-actin) is required for cell movement, contraction, and a variety of physiological processes associated with cell membrane remodeling (43). Viral attachment to host cell membrane receptors is an initial infection step that has been functionally linked to cell membrane deformation associated with membrane recruitment, thereby promoting viral entry (44, 45). Although the identities of mammalian and avian cell receptors used by corresponding HEVs remain unclear, CaHEV-induced Cofilin activation and regulation of the actin cytoskeleton may be involved in virion internalization. Based on the visualization of actin filaments using FITC-labeled phalloidin, triggering endocytosis-mediated internalization in CaHEV-infected cells and ap237-inoculated LMH-1A2 cells induced typical actin rearrangements involving the formation of lamellipodia, filopodia, and stress fibers (Fig. 11A). These changes were most markedly apparent by 15 and 45 min after an endocytosis-inducing temperature switch (Fig. 11A). The quantification of lamellipodia, filopodia, and stress fibers in CaHEV-infected cells at different time points is shown in Fig. 11B. Thus, CaHEV-induced Cofilin activation triggered by Rap1b and Talin-1 activation apparently induced actin remodeling that potentially facilitated CaHEV infection. In addition to the actin pattern, the subcellular localization of the ap237 protein was investigated, and the results indicated that the ap237 protein entered cells and then moved from the cell membrane to the cytoplasm after the temperature switch. Once appearing in the cytoplasm, ap237 protein migrated to the area surrounding the nucleus; this result is consistent with known events resulting from actin rearrangement (Fig. 11C), which suggests that actin remodeling contributes to CaHEV capsid internalization.

Conversely, by employing the F-actin polymerization inhibitor cytochalasin D (CytoD) to block actin remodeling, dose-dependent reductions in internalized virions and viral capsids were observed in CaHEV-infected cells and ap237-incubated cells, respectively (Fig. 11E and F), whereas the dose of CytoD used to block F-actin polymerization demonstrated minimum cytotoxicity (Fig. 11D). Moreover, F-actin staining revealed that CytoD-induced inhibition of F-actin polymerization suppressed the formation of lamellipodia and stress fibers, leading to membrane retention of capsid protein in permissive cells (Fig. 11G). These results further suggested that actin rearrangement may participate in CaHEV internalization. In summary, our results suggested that that the CaHEV capsid protein interacts with Rap1b to enhance Rap1b activation, which induces membrane recruitment of Talin-1 and leads to activation of Integrin-FAK-Cofilin cascades. As a key regulator of actin kinetics, Cofilin activation results in actin and membrane remodeling that facilitates CaHEV virion internalization.

## DISCUSSION

Big liver and spleen (BLS) disease in chickens, which is a typical feature of avian HEV infection, is characterized by a sudden drop in egg production accompanied by spleen enlargement and ovarian histopathological changes, thereby accounting for significant economic losses in the poultry industry (46). However, the mechanisms underlying avian HEV infection and pathogenesis remain unclear. In this study, the Rap1b protein was identified as a CaHEV capsid-interacting candidate in chicken liver tissues, and our results suggest that Rap1b activation and its downstream pathways were potentially hijacked by CaHEV to facilitate virion internalization into permissive cells.

Rap1b is a small GTPase with multiple biological functions, such as maintaining cell-to-cell adhesion, cell morphology, and membrane transport (26). Generally, prenylated Rap1b is activated by GEFs such as the chaperone protein SmgGDS (26, 47), thus conferring GTP loading status. In this study, SmgGDS-associated GEF activity in LMH-1A2 cells was directly correlated with enhanced Rap1b activation after CaHEV infection or ap237 inoculation in LMH-1A2 cells. Nevertheless, in the presence of CaHEV or ap237, Rap1b activation depends on PKA or SmgGDS. Although it remains unclear how interactions between viral capsids and Rap1b lead to enhancement of PKA- and SmgGDS-dependent Rap1b activation, these results underscore the importance of Rap1b in virus infection. Concurrently, enhancement of Rap1b activation induced by CaHEV caused membrane recruitment of the downstream effector molecules Rap1b-GTP interacting adapter protein (RIAM) and Talin-1, both of which are known to participate in inside-out integrin-based signaling. However, blocking virus or capsid protein attachment and internalization using anti-CaHEV antibodies suppresses RIAM membrane redistribution, thereby further suggesting that an interaction between viral capsids and Rap1b triggers downstream RIAM and Talin-1 activation during CaHEV infection.

RIAM could act as a bridge that transfers a signal from activated Rap1b to Talin-1 through the Rap1b-RIAM-Talin-1 signaling pathway. Once Talin-1 is activated, it anchors to the cell membrane to bind with cytoplasmic tails of integrins (28, 29), which leads to an inside-out activation of integrins (29–31, 38); this process is different from that of other types of membrane receptors that receive extracellular signals through recognition of extracellular ligands (e.g., cytokines). Increasing evidence suggests that viruses may harness host integrin-induced cytoskeleton-based processes to enter host cells (34, 48). Integrin α5/β1 has been shown to act as a potential receptor for several viruses, such as foot-and-mouth disease virus (37), adeno-associated virus type 2 (35), and subtype B avian metapneumovirus (36). Moreover, integrin α5/β1 triggers the FAK-RAC1&CDC42-PAK-LIMK-Cofilin pathway, and cytoskeletal rearrangement appears to promote porcine hemagglutinating encephalomyelitis virus infection of permissive cells (34). Our results suggest that the internalization of CaHEV triggered the inside-out activation of integrin α5/β1 along with downstream cascade signaling, including Cofilin, as evidenced by a direct interaction between membrane-anchored Talin-1 and the cytoplasmic tail of integrin α5/β1.

Membrane protrusion, invagination, and fusion along with corresponding membrane changes are involved in many physiological processes of cells, such as metabolism, signal transduction, and cell migration (49). Due to dynamic structural membrane changes associated with different F-actin states (50), mechanical forces resulting from filopodia formation or stress fiber-induced cell membrane remodeling may promote the internalization of membrane-bound virions. Phosphorylation and dephosphorylation of the actin regulatory protein Cofilin are known to be directly responsible for regulating the formation and recycling of actin filaments and are regulated by the upstream kinase LIMK1 in response to signals that trigger F-actin-associated membrane deformation (51, 52). Accordingly, CaHEV-induced cytoskeletal rearrangements, as reflected by changes in lamellar lipoproteins, filopodia, and stress fibers, were diminished by treatment with the F-actin inhibitor CytoD, further demonstrating that Rap1b-related cell membrane transport involved integrin α5/β1 activation and played an important role in CaHEV infection. Although integrin pathway-associated signals downstream of Rap1b activation signals have been extensively investigated as regulators of cytoskeleton-associated activities (53, 54), few reports link Rap1b with viral infections. Nevertheless, our findings suggest that CaHEV may exploit cytoskeleton regulation-related physiological processes to gain entry into host cells.

Macropinocytosis, which is one of the clathrin-independent endocytic routes, is an actin-dependent process initiated from surface membrane ruffles that give rise to large endocytic vacuoles called macropinosomes, and involves actin-mediated membrane ruffling of the plasma membrane (55). Moreover, induction of macropinocytosis by oncogenic Ras members has been characterized in the setting of cancer cells endogenously expressing oncogenic Ras (56). Therefore, it appears that the interaction between ap237 and Rap1b induced macropinocytosis to facilitate avian HEV virion uptake by permissive cells. As a previous report suggested that human hepatitis E virus enters liver cells through receptor-dependent clathrin-mediated endocytosis (57, 58), it is possible that both Rap1b-induced macropinocytosis and clathrin-mediated endocytosis both contributed to the entry of avian HEVs.

It has long been known that HEV virions exist in two distinct forms, namely, a “naked form” and a “quasi-enveloped form,” whereas different virion forms utilize distinct entry mechanisms to infect susceptible cells, as evidenced using an *in vitro* HEV-3 Kernow-C1 p6 cell culture system (59). In the scenario of avian HEV, although no conclusive evidence could confirm that the existence of quasi-enveloped virions as an *in vitro* cell culture system for avian HEV is not sufficiently robust for large-scale virion production, it is possible that avian HEV has a “quasi-enveloped” virion form. This form should be similar to that of mammalian HEV, since the PSAP motif (approximately aa95-98) in mammalian HEV-ORF3 required for HEV virion release from infected cells is conserved in avian HEV-ORF3 and plays the same role in virion release (60–62). However, due to the available source for obtaining virus stock in our study, the CaHEV virions used to inoculate LMH-1A2 were collected from fecal samples, which are supposed to be naked. Therefore, whether the ORF2-Rap1b interaction is also involved in the entry of susceptible cells by quasi-enveloped CaHEV virions requires further investigation.

An early report demonstrated that the small GTPases Rab5 and Rab7, which are commonly involved in virus trafficking through the early endosome (Rab5) and the late endosome (Rab7), were involved in the infection of susceptible cells by quasi-enveloped HEV virions other than naked virions (59), whereas HEV-VLPs were internalized via a dynamin-2-, clathrin-, and membrane cholesterol-dependent pathway (57, 58). Similar to Rap1b, Rab5, and Rab7 are the GTPases belonging to the Ras GTPase superfamily that broadly control budding, uncoating, motility, and fusion of vesicles in most cell types (63); however, the role of Rap1b in mammalian HEV (naked form) infection requires further investigation. As heparan sulfate proteoglycan (HSPG) has been shown to be involved in the cell attachment of mammalian HEV virus-like particles and preincubation of HEV with heparan sulfate blocks HEV infectivity (21, 59), it is possible that Rap1b or other Ras GTPases participate in steps downstream of virion-receptor binding to mediate internalization of naked mammalian HEV virions.

In conclusion, the present study demonstrated cellular uptake of naked CaHEV infection appears to depend on Rap1b-associated cytoskeletal rearrangement and such rearrangement depends on activation of Rap1b-GTP, which requires interaction between the CaHEV capsid protein and Rap1b. Activated Rap1b triggers sustained membrane recruitment of Talin-1 and induces the activation of integrin α5/β1, which further leads to downstream activation of integrin-FAK-cofilin cascades and ultimately to actin rearrangement and membrane remodeling that promotes the internalization of CaHEV virions. In summary, our research suggests novel roles of the entry mechanism and pathogenesis of avian HEV and potentially provides new insight into the entry mechanism of mammalian HEV.

## MATERIALS AND METHODS

### Cell, virus, and chemicals.

The generation of a hepatocellular carcinoma cell line (LMH cells) with stable expression of OATP1A2 (LMH-1A2) has been previously described (19). LMH-1A2 cells were grown in Dulbecco’s modified Eagle’s medium (Gibco, Carlsbad, CA, USA) with 10% fetal bovine serum (Gibco) supplemented with 100 U/mL penicillin and 100 μg/mL streptomycin (Thermo Fisher Scientific, Waltham, MA, USA) at 37°C with 5% CO2. The Chinese avian HEV strain (CaHEV, GenBank accession number GU954430), initially isolated from chicken bile samples, was produced using a previously described method and stored at −80°C (19). The RNA copy numbers of the CaHEV viral stock were quantified with quantitative real-time PCR (qPCR) and used to inoculate LMH-1A2 cells at 20 copies per cell throughout this study. For the chemical agents, CytoD was obtained from Selleck Chemicals (Houston, USA). Forskolin and H-89 were obtained from Beyotime Biotechnology (Beijing, China). ATN-161 was obtained from Sigma–Aldrich (St. Louis MO, USA).

### Recombinant expression of viral proteins.

The production of truncated CaHEV-ORF2 protein ap237 (aa313-aa549) has been previously described (19). Recombinant CaHEV-ORF3 protein (ORF3^CaHEV^) fused with a 6×His tag was produced by ligating the cDNA of CaHEV-ORF3 to the pET21b (Omega Bio-Tek, Noclos, GA, USA) vector and transforming it into TransB(*DE3*) competent cells (TransGen Biotech, Beijing, China). Protein expression was induced with 0.5 M isopropyl-beta-d-thiogalactoside (IPTG, Sigma–Aldrich) at 28°C for 8 h before centrifugation and sonication. Next, the recombinant ORF3^CaHEV^ protein from the supernatant of the bacterial lysate was purified using Ni^2+^ affinity chromatography (TransGen Biotech) and eluted with elution buffer as previously described (19). The purified ap237 and ORF3^CaHEV^ proteins were quantified using a Pierce BCA Protein assay kit (Thermo Fisher Scientific) according to the manufacturer's instructions.

### Antibodies.

Antibodies against Flag, Rap1b, PKA, SmgGDS, RIAM, Talin-1, FAK, CDC42, RAC1, PAK1, Cofilin, Na^+^/K^+^ ATPase, and GAPDH were obtained from ProteinTech (Wuhan, China). Antibodies against phospho-PKA, phospho-FAK, Src, phospho-Src, phospho-PAK1, LIMK1, phospho-LIMK1, and phospho-Cofilin were obtained from Cell Signaling Technology (Boston, MA, USA). Antibodies against Integrin α5 and Integrin β1 were obtained from Boster BioTech (Wuhan, China). Cy3-conjugated goat antimouse IgG (H+L), Cy3-conjugated goat antirabbit IgG (H+L), Alexa Fluor 488-conjugated goat antimouse IgG (H+L), Alexa Fluor 488-conjugated goat antirabbit IgG (H+L), and Cy3-conjugated goat anti-rabbit IgG (H+L) were obtained from Jackson ImmunoResearch (West Grove, PA, USA). FITC-conjugated phalloidin was obtained from Solarbio Life Science (Beijing, China). The CaHEV-specific monoclonal antibody (Mab) named Mab-1B5 was produced previously with truncated CaHEV ORF2 protein (ap268) as the immunogen (64). CaHEV convalescent chicken sera were obtained from our previous CaHEV infection experiment (19). Enrichment of ap237-specific polyclonal IgY from CaHEV convalescent chicken sera was conducted by passing sera from CNBr-activated Sepharose 4B resin (GE Healthcare) conjugated with recombinant ap237 protein according to the manufacturer’s instructions. The antibody concentration was quantified using an Epoch-BioTek Microplate Reader (USA) based on the absorbance value at a wavelength of 280 nm. Normal chicken IgY was purchased from Sigma–Aldrich and used as a control for ap237-specific polyclonal IgY. Normal mouse IgG was purchased from Santa Cruz Biotechnology (Santa Cruz, CA, United States) and used as a control for the immunoprecipitation assay.

### Ethics statement and animal experiments.

Eight-week-old SPF chickens were purchased from Dashuo Biotech (Chengdu, Sichuan, China) and fed in an environment with 12 h of darkness and 12 h of light. All animal experiments used in this study were approved by the Committee on Ethical Use of Animals of Northwest A&F University, care of animals was conducted strictly according to the guidelines of Northwest A&F University Institutional Committee, and all efforts were made to minimize suffering of the animals. To analyze the link between Rap1b and CaHEV infection, 6 chickens were inoculated intravenously with 1 mL virus stock (3.5 × 10^7^ copies/mL), and 6 chickens were inoculated with 1 mL PBS as a negative control. Chicken sera and fecal samples were collected weekly for monitoring viremia and fecal shedding of virus by qPCR. Forty-two days after virus inoculation, liver tissue samples of all chickens were collected and homogenized using liquid nitrogen for RNA extraction and Western blot analysis of target proteins and genes.

### Entry kinetics of CaHEV in cells.

To evaluate CaHEV entry into LMH-1A2 cells, cells were first prechilled at 4°C for 10 min and then incubated with CaHEV (20 copies per cell) or ap237 protein (750 nM) at 4°C for 2 h or 1 h, respectively. Free virion and ap237 protein were removed by washing cells with ice-cold PBS. Next, cells were transferred and maintained at 37°C for the indicated times to trigger virion internalization. Noninternalized virions were removed using citrate buffer (40 mM sodium citrate, 10 mM KCl, and 135 mM NaCl, pH 3.1) for 30 s, while noninternalized ap237 protein was removed with 0.25% trypsin for 10 s. Subsequently, internalized CaHEV or ap237 protein was evaluated by harvesting cells for quantification of viral RNA using qPCR or quantification of ap237 protein using Western blotting, respectively. Cells pretreated with chemical agents at the indicated concentration or transfected with siRNAs to block stages involved in virion/ap237 protein attachment and internalization were harvested with the same protocol mentioned above for qPCR or Western blotting.

### RNA extraction, reverse transcription, and qPCR.

Homogenized chicken liver tissues and other cell samples were harvested using RNAiso Plus (TaKaRa, Dalian, China) for RNA extraction according to the manufacturer’s instructions. The cDNAs were reverse transcribed using the PrimeScript RT reagent kit (TaKaRa) and subjected to real-time quantitative PCR (qPCR) using 2X Real Star SYBR Green Mix (GenStar, Beijing, China). Transcripts of glyceraldehyde-3-phosphate dehydrogenase (GAPDH) from corresponding samples were included as the housekeeping gene to normalize the RNA inputs. Calculation of the relative expression level of target genes was calculated based on the 2^−ΔΔCt^ method. For absolute quantification qPCR, a previously described plasmid pMD-18T-ORF3 was used for standard curve and RNA copy number calculation of CaHEV (19). All primers used in this study and the corresponding sequences are listed in Table 1.

**TABLE 1:** Primer sequences of qPCR used in this study

Primer name	Sequences (5′–3′)
Rap1b-gallus-qF	AGTGTGACTTGGAAGATGAAAGAGT
Rap1b-gallus-qR	CTGTTA ATTTGYCGCACWARGTCAT
ORF3-avian-qF	TATGTGCTGCGGGGTGTCAA
ORF3-avian-qR	CATCTGGTACCGTGCGAGTA
GAPDH-gallus-qF	AAACTCATTGTCATACCAGG
GAPDH-gallus-qR	ATACACAGAGGACCAGGTTG

### Western blotting.

Cell samples were washed twice using ice-cold PBS and lysed with NP40 buffer (Beyotime) containing 1 mM protease inhibitor (Beyotime) or 20 mM phosphatase inhibitor cocktail I (Targetmol, Shanghai, China) on ice for 30 min. The cell lysate was cleared by centrifugation at 13,000 *g* at 4°C for 10 min, and the supernatants were harvested for further experiments. A cell membrane protein and cytoplasmic protein extraction kit (Beyotime) was used for the extraction of cell membrane and cytoplasmic proteins according to the manufacturer’s instructions. All harvested samples were quantified with a Pierce BCA Protein assay kit (Thermo Fisher Scientific).

The extracted protein samples were harvested with 5 × SDS–PAGE sample buffer (GenStar), boiled for 10 min to denature proteins, separated by sodium dodecyl sulfate-polyacrylamide gel electrophoresis (SDS–PAGE), and electrotransferred to a 0.22-μm polyvinylidene fluoride (PVDF) membrane. After blocking with blocking buffer (5% skimmed milk powder dissolved in PBS containing 0.25% Tween 20) at 37°C for 1 h, the membranes were probed with the indicated antibodies diluted in blocking buffer for another 1 h. The interaction between antibodies and corresponding targets was visualized using HRP-labeled secondary antibodies and ECL Substrate (Solarbio). The chemiluminescent signal was recorded using a ChemiDoc MP Image System (Bio–Rad Laboratories) and analyzed using ImageLab software (Version 5.1, Bio–Rad Laboratories). GAPDH and Na^+^/K^+^ ATPase were used as loading controls for total and membrane protein.

For the Western blotting-based evaluation of activated forms of Rap1b (Rap1b-GTP), CDC42 (CDC42-GTP), and RAC1 (RAC1-GTP) from the indicated samples, corresponding cells or tissue samples were subjected to target protein enrichment using the Active Rap1 Detection Kit (Cell Signaling Technology), Active Cdc42 Detection Kit (Cell Signaling Technology), and Active RAC1 Detection Kit (Cell Signaling Technology), respectively, according to the manufacturer's instructions. Enriched proteins were subjected to SDS–PAGE and Western blotting as mentioned above.

### Pull-down assay.

To confirm the interaction between the capsid of CaHEV and Rap1b, CNBr-activated Sepharose 4B resin (GE Healthcare, Chicago, IL, USA) was washed with a cold solution of 1 mM HCl and conjugated to ap237 protein following the manufacturer’s instructions at a ratio of 1 mL Sepharose 4B to 4 mg ap237 protein. Subsequently, the extracted liver tissue sample was mixed with ap237-conjugated 4B resin and incubated at 4°C for 6 h. Then, the interaction complexes from ap237-conjugated 4B resin were eluted and subjected to SDS–PAGE followed by sliver staining using a sliver stain kit (Solarbio). Western blotting using Rap1b-specific polyclonal antibodies was conducted to verify the existence of Rap1b. Sepharose 4B resin conjugated to recombinant ORF3^CaHEV^ protein served as an irrelevant control.

### RNA interference.

LMH-1A2 cells were seeded in 12-well plates at a density of 1 × 10^5^ cells/mL in DMEM containing 10% FBS and cultured overnight. After the cell confluence reached 50%–60%, transfection of the indicated siRNAs was carried out using Lipofectamine RNAiMAX reagent (Thermo Fisher Scientific) in accordance with the manufacturer’s protocol. The siRNA and corresponding sequence used in this study are listed in Table 2.

**TABLE 2:** siRNA target sequences used in this study

siRNA	Sequences (5′–3′)
si-Rap1b	AGGAATATTTGTTGAAAAATACG
si-SmgGDS	GGGA ACATTACGAATGTTAATAG
si-RIAM	TGGTGTCTATATGAAGTCTATCC
si-Talin-1	CTCAATGTCACTGAGAACATATT
si-Src	CACTTTCGTGGCTCTCTATGACT
si-FAK	AGCAATCAAAACATGTAAAAACT

### Immunofluorescence assay.

LMH-1A2 cells were seeded in 24-well plates with cover glass at a density of 4 × 10^4^ cells/well for 12 h. Then, cells with the indicated treatments were washed with PBS and fixed with 4% paraformaldehyde at RT for 15 min. After washing with fresh PBS, cells were permeabilized with PBS containing 0.25% Triton X-100 and further blocked with PBS containing 2% bovine serum albumin for 1 h at 37°C. Next, blocking buffer-diluted antibodies were used to probe the cells, and specific binding between antibodies and their targets was visualized using secondary antibodies with corresponding conjugates. F-actin and cellular nuclei were counterstained with FITC-conjugated phalloidin (Solarbio Life Science) and 4,6-diamidino-2-phenylindole (DAPI; Thermo Fisher Scientific). Cell slides were observed with a Leica SP8 confocal system (Leica Microsystems, Wetzlar, Germany). All images were captured and processed using Leica Application Suite X (Leica Microsystems).

### Coimmunoprecipitation assay (Co-IP assay).

Interactions between target proteins and potential interacting partners were analyzed using a Co-IP assay. Briefly, after washing Protein G Dynabeads (Thermo Fisher Scientific) with NP40 buffer, the Dynabeads were precoated with 6 μg antibodies against Flag, ap237, Rap1b, integrin α5, or integrin β1 at 4°C for 6 h. Next, antibody-precoated beads were added to the total or membrane protein extract and incubated for another 6 h at 4°C. After washing the beads with NP40 buffer 3 times, the immune complex from the beads was eluted using 1 × SDS–PAGE sample buffer, boiled for 10 min, and further subjected to SDS–PAGE and Western blotting with the corresponding antibodies.

### Cytotoxicity assays.

The cytotoxicity of chemicals to LMH-1A2 cells was determined using a CCK-8 Kit (Beyotime). Briefly, LMH-1A2 cells were seeded in 96-well plates at a density of 5 × 10^3^ cells/well and grown overnight. Next, the cells were treated with different chemicals at different concentrations at 37°C for 1 h and then changed to mixtures of 90 μL fresh DMEM containing drugs and 10 μL CCK-8 solution for culturing at 37°C for another 2 h. The absorbance values of each treatment group at 450 nm were measured by an Epoch-BioTek Microplate Reader (USA). Equal amounts of solvents (DMSO or water) for dissolving chemicals were added to cells as solvent controls.

### Evaluation of the membrane distribution of RIAM and Talin-1.

To determine the membrane distribution of RIAM and Talin-1, LMH-1A2 cells were infected with CaHEV or treated with ap237 protein at 4°C for 2 h or 1 h of binding, respectively. Next, the cells were transferred to 37°C to trigger endocytosis-mediated internalization of virus or protein and subjected to the extraction of membrane proteins and cytoplasmic proteins. After washing the cells with PBS, the plasma membrane and cytoplasm were harvested and subjected to Western blotting using anti-RIAM and anti-Talin-1 antibodies. GAPDH and Na^+^/K^+^ ATPase served as protein controls for plasma proteins and membrane proteins, respectively.

### Statistical analysis.

All immunofluorescence assay, confocal microscopy, and Western blotting images were quantified using Leica Application Suite X (Leica Microsystems) and ImageJ software (USA) for statistical analysis. The statistical significance of all line charts and histogram-associated data within each group was performed using GraphPad Prism version 5.0 (GraphPad Software, San Diego, CA, USA). Statistical significance was determined using either Student's *t* test for comparisons of two groups or one-way analysis of variance (ANOVA) for comparisons among more than two groups. A two-tailed *P* value < 0.05 was considered statistically significant.
